# Supplementary figures and images for: Is maternal weight gain between pregnancies associated with risk of large-for-gestational age birth? Analysis of a UK population-based cohort
Source: BMJ Open. 2019 Jul 9;9(7):e026220. doi: 10.1136/bmjopen-2018-026220 (PMC6615839; doi:10.1136/bmjopen-2018-026220)

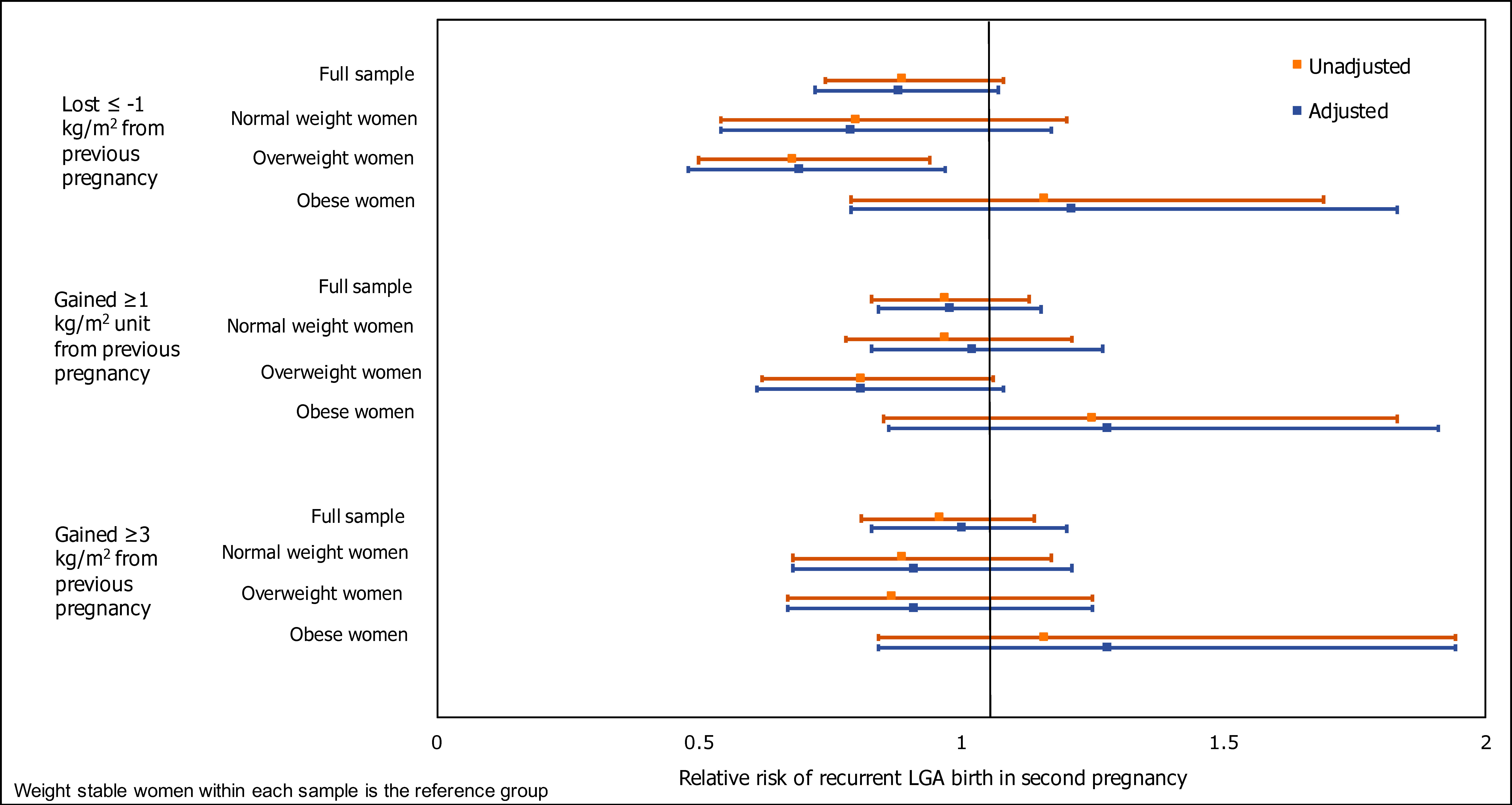

Supplement: Supplementary data [file bmjopen-2018-026220supp001.jpg]

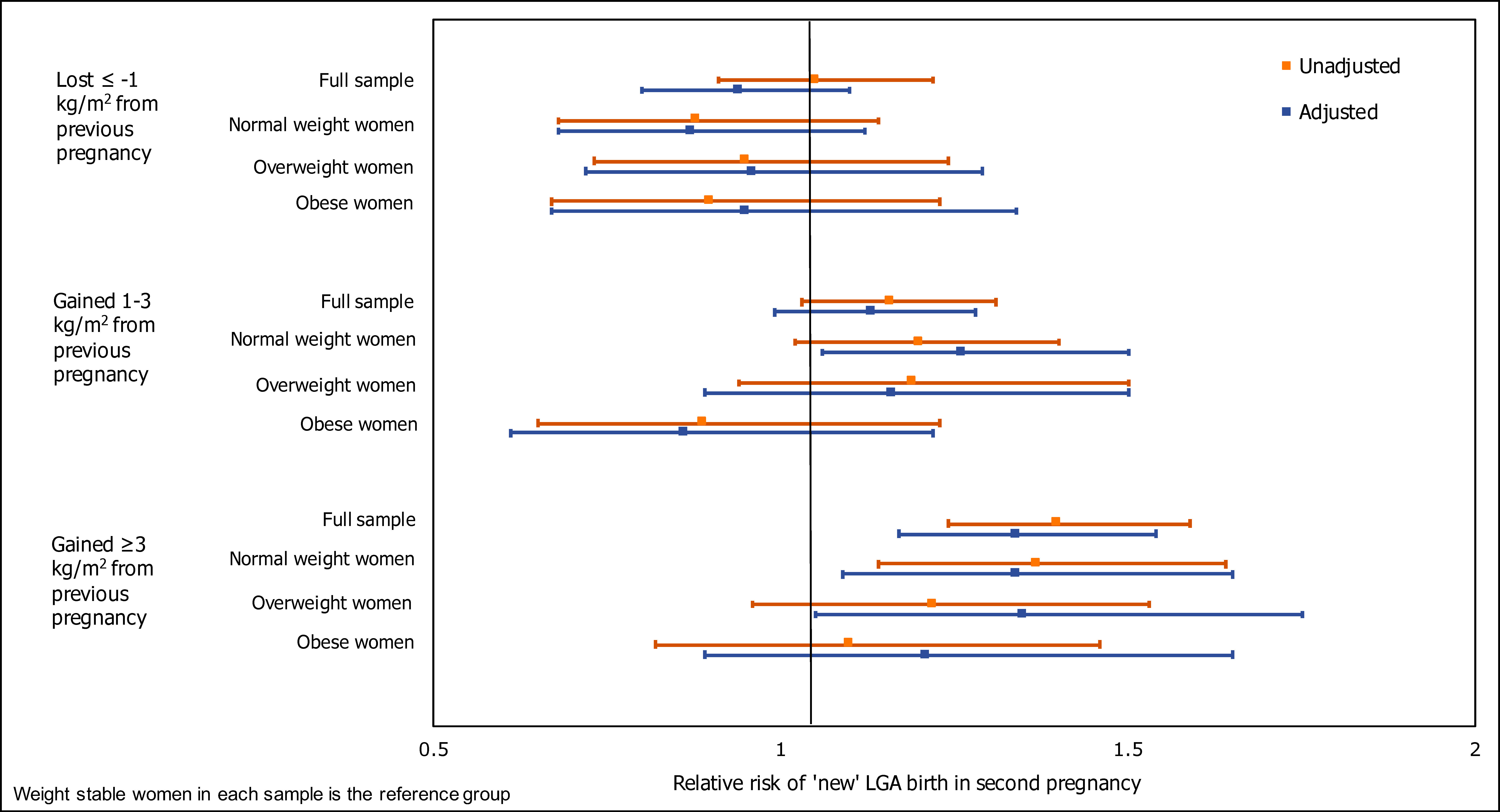

Supplement: Supplementary data [file bmjopen-2018-026220supp002.jpg]
