# Supplementary material for: Is maternal weight gain between pregnancies associated with risk of large-for-gestational age birth? Analysis of a UK population-based cohort
Source: BMJ Open. 2019 Jul 9;9(7):e026220. doi: 10.1136/bmjopen-2018-026220 (PMC6615839; doi:10.1136/bmjopen-2018-026220)
Supplement: Author's manuscript [file bmjopen-2018-026220.draft_revisions.pdf]

BMJ Open is committed to open peer review. As part of this commitment we make the peer review history of every article we publish publicly available.

When an article is published we post the peer reviewers' comments and the authors' responses online. We also post the versions of the paper that were used during peer review. These are the versions that the peer review comments apply to.

The versions of the paper that follow are the versions that were submitted during the peer review process. They are not the versions of record or the final published versions. They should not be cited or distributed as the published version of this manuscript.

BMJ Open is an open access journal and the full, final, typeset and author-corrected version of record of the manuscript is available on our site with no access controls, subscription charges or pay-per-view fees (http://bmjopen.bmj.com).

If you have any questions on BMJ Open's open peer review process please email info.bmjopen@bmj.com

BMJ Open

Is maternal weight gain between pregnancies associated with risk of large-for-gestational age birth? Analysis of a UK population-based cohort

Journal:	*BMJ Open*
Manuscript ID	bmjopen-2018-026220
Article Type:	Research
Date Submitted by the Author:	24-Aug-2018
Complete List of Authors:	Ziauddeen, Nida; University of Southampton, Academic unit of Primary Care and Population Sciences, Faculty of Medicine<br>Wilding, Sam; University of Southampton, Academic unit of Primary Care and Population Sciences, Faculty of Medicine<br>Roderick, Paul; University of Southampton, Academic unit of Primary Care and Population Sciences, Faculty of Medicine<br>Macklon, Nick; University of Copenhagen, Zealand University Hospital, Department of Obstetrics and Gynaecology; London Women's Clinic<br>Alwan, Nisreen; University of Southampton, Academic unit of Primary Care and Population Sciences, Faculty of Medicine; University of Southampton and University Hospital Southampton NHS Foundation Trust, NIHR Southampton Biomedical Research Centre
Keywords:	obesity, pregnancy, pregnancy outcome, weight gain, birth weight

SCHOLARONE™
Manuscripts

For peer review only

Is maternal weight gain between pregnancies associated with risk of large-for-gestational age birth? Analysis of a UK population-based cohort

Nida Ziauddeen[1], Sam Wilding[1], Paul J. Roderick[1], Nicholas S. Macklon[2,3], Nisreen A. Alwan[1,4]

[1] Academic Unit of Primary Care and Population Sciences, Faculty of Medicine, University of Southampton, Southampton, UK

[2] Department of Obstetrics and Gynaecology, University of Copenhagen, Zealand University Hospital, Roskilde 4000, Denmark

[3] London Women's Clinic, 113-115 Harley Street, London W1G 6AP

[4] NIHR Southampton Biomedical Research Centre, University of Southampton and University Hospital Southampton NHS Foundation Trust, Southampton SO16 6YD, UK

Author emails: N.Ziauddeen@soton.ac.uk, S.A.Wilding@soton.ac.uk, pjr@soton.ac.uk, nism@regionsjaelland.dk, N.A.Alwan@soton.ac.uk

Corresponding author: Nida Ziauddeen

Academic Unit of Primary Care and Population Sciences, Faculty of Medicine, Mailpoint 805, Level C (AC22), South Academic Block, Southampton General Hospital, Tremona Road, Southampton SO16 6YD, UK

Email: N.Ziauddeen@soton.ac.uk

Telephone number: 023 81 206287

Keywords: obesity, pregnancy, pregnancy outcome, weight gain, birth weight

Word count: 3924 words

For peer review only - http://bmjopen.bmj.com/site/about/guidelines.xhtml

Abstract

Objective: Maternal obesity during pregnancy increases the risk of large-for-gestational age (LGA) infant and childhood obesity. The aim was to investigate the association between maternal weight change between subsequent pregnancies and risk of having a LGA baby.

Design: Population-based cohort.

Setting: Routinely collected antenatal healthcare data between January 2003 and September 2017 at University Hospital Southampton, England.

Participants: Records of women with their first two consecutive singleton live-birth pregnancies were analysed (n=15940).

Primary outcome measure: Risk of LGA, recurrent LGA and 'new' LGA births in the second pregnancy.

Results: Of the 15940 women included, 16.0% lost and 47.7% gained weight (≥1 body mass index (BMI) unit) between pregnancies. A lower proportion of babies born to women who lost ≥1 BMI unit (12.4%) and remained weight stable between -1 to 1 BMI unit (11.9%) between pregnancies were LGA compared to 14.5% in women who gained ≥1 BMI unit (adjusted odds ratio (aOR) of LGA for weight gain compared to weight stable 1.24, 95% CI 1.11 to 1.39, P<0.001). Normal- and over-weight women who gained weight were at increased risk of LGA after having a non-LGA baby in the first pregnancy (aOR 1.37, 95% CI 1.16 to 1.61, p<0.001 in normal weight and aOR 1.30, 95% CI 1.02 to 1.65, p=0.03 in overweight). Overweight women who had a LGA baby in the first pregnancy were at lower risk of LGA in the second pregnancy if they lost ≥1 BMI unit (aOR 0.44, 95% CI 0.23 to 0.85, p=0.02).

Conclusions: Losing weight after an LGA birth in overweight women reduces the risk of subsequent LGA in the next pregnancy, while gaining weight increases its risk in women with no previous history of LGA. Preventing weight gain between pregnancies is an important prevention measure to achieve better maternal and offspring outcomes.

Article summary

Strengths and limitations of this study

- Utilises data from a large population-based cohort including women from all socioeconomic backgrounds
- Data is collected as part of routine care during pregnancy
- Objective measurement of exposure and outcome
- Self-reported data for confounders
- Lack of information on weight gain during pregnancy

For peer review only - http://bmjopen.bmj.com/site/about/guidelines.xhtml

Introduction

Maternal obesity has shown a significant increase over time, having more than doubled in England between 1989 to 2007 (7.6% to 15.6%), with the rate of normal weight pregnancies showing a 12% decrease from 65.6% to 53.6%[1]. Maternal overweight and obesity is a key risk factor for maternal and foetal outcomes. It also increases the risk of long-term health problems in the child including obesity, cardiovascular disease, diabetes and cognitive and behavioural disorders[2]. Change in maternal body mass index (BMI) between pregnancies could modify the risk in the subsequent pregnancy.

Birthweight is a key early life predictor of long-term health outcomes such as obesity and cardiovascular disease[3]. The incidence of large-for-gestational age (LGA) birth, defined as >90th percentile weight for gestational age, has increased over time in high-income countries[4,5]. A key risk factor for LGA birth is gestational diabetes mellitus[8], the incidence of which has also increased over time[6,7]. LGA has been found to be associated with childhood obesity prevalence at age 7 years[9,10] and into adulthood[11-13]. Offspring of mothers with gestational diabetes have increased risk of overweight and obesity at age 7 years[14,15]. Maternal obesity is a known risk factor for both gestational diabetes and LGA birth[16].

Birthweight increases with parity such that the first-born infant on average has the lowest birthweight and the birthweight of subsequent infants increases[17-19] up to the fourth pregnancy[20]. However, birthweight was found to decrease with parity for women who had short intervals between pregnancies and the increase in birthweight with parity was higher in women with long intervals[20]. Women who returned to their pre-pregnancy weight before the next conception had subsequent born infants who weighed less than infants of women who retained or gained weight between pregnancies[20]. Women who lost at least six kilograms by their second pregnancy had a smaller average increase in birthweight compared to women who gained ten kg or more[18].

A large US study showed that women were at an increased risk of having an LGA baby in the second pregnancy if their pre-pregnancy BMI category increased towards overweight or obese from first to second pregnancy regardless of their BMI category in first pregnancy, except in underweight women whose weight increases to become within the normal range. In this study, overweight and obese women who dropped BMI category by their second pregnancy remained at an increased risk of LGA birth, but had a lower risk compared to women whose BMI category increased between pregnancies[21].

Another US-based study showed that inter-pregnancy weight gain of ≥2 BMI units in women who were obese in their first pregnancy was associated with increased risk of LGA. Weight loss of ≥2 BMI units was associated with lower risk compared to the reference group of weight maintained between 2 BMI units adjusted for LGA birth in previous pregnancy and other confounders[22].

Three studies assessed weight change between first and second pregnancy in relation to ‘new’ LGA incidence in the second pregnancy, stratified by BMI category (< or ≥25)[23-25]. Two studies found a reduced risk of ‘new’ LGA with between pregnancy weight loss of >1 BMI unit and an increased risk with modest (1-3 BMI units) and large (≥3 BMI units) weight gain. The effect remained in both BMI categories after stratification (< or ≥25) but was stronger in women with a healthy first pregnancy BMI (<25kg/m^2). The third study only found an increased risk in normal weight women who gained ≥4 BMI units between pregnancies and no association in overweight women[25].

Only one published study has examined the risk of recurrent LGA (in both first and second pregnancies) in relation to maternal weight change between pregnancies [26]. The study, which was conducted in Aberdeen, Scotland, included 24520 women of which 813 women had LGA births in both pregnancies, and found that inter-pregnancy weight gain (≥2 BMI

For peer review only - http://bmjopen.bmj.com/site/about/guidelines.xhtml

units) was associated with increased risk of recurrent LGA, while weight loss (≥2 BMI units) was found to be protective. Women with healthy weight (BMI <25kg/m^2) were at increased risk of recurrent LGA on gaining weight whereas overweight women (BMI ≥25kg/m^2) were at reduced risk of recurrent LGA on losing weight [26].

We aimed to investigate the association of the incidence of LGA, recurrent LGA and 'new' LGA births in the second pregnancy with maternal change in BMI between the first and second pregnancies in a population-based cohort in the South of England.

Methods

This is a population-based cohort of prospectively collected routine healthcare data for antenatal care between January 2003 and September 2017 at University Hospital Southampton, Hampshire, UK. This included all women delivering at this hospital, which is a regional centre for maternity care in and around Southampton. Records of women with their first two consecutive singleton live birth pregnancies were included. Records with unfeasible weight, height and gestational age values were excluded.

Exposure assessment

Maternal weight in kilograms was measured by a midwife at the first antenatal (booking) appointment of each pregnancy, which is recommended to take place between 8 to 12 weeks gestation in the UK, according to the National Institute for Health and Care Excellence Guidelines [27]. Any woman who had a booking appointment at or after 24 weeks of pregnancy was excluded. Height was self-reported. BMI was calculated as weight (in kg) divided by height (in metres) squared.

BMI was categorised as underweight (BMI <18.5 kg/m^2), normal weight (18.5 to 24.9 kg/m^2), overweight (25.0 to 29.9 kg/m^2) and obese (≥30 kg/m^2). Change in BMI was calculated as the difference in BMI measured at the booking appointments of the first two consecutive live birth pregnancies for each woman. This change in BMI was then categorised as weight loss (≥1 BMI unit), weight stable (-1 to 1 BMI unit) and weight gain (≥1 BMI unit).

Outcome assessment

Birthweight (grams) was measured by midwives at birth. Gestational age was based on a dating ultrasound scan which usually takes place by 13 weeks gestation[27]. Birthweight centiles was calculated using reference values for England and Wales provided in the most recently released data[28]. Large-for-gestational age was defined as >90th percentile weight for gestational age. This was only defined for babies born between 24 to 42 weeks gestation as reference values only exist for these gestational ages.

Covariables

Maternal date of birth is recorded at the booking appointment and converted to age on extraction of the dataset to maintain anonymity. Highest maternal educational qualification was self-reported and categorised as primary, secondary, college, undergraduate, postgraduate, graduate and none. For the purposes of this analysis, this was condensed to three categories - secondary (GCSE) and under, college (A levels) and university degree or

For peer review only - http://bmjopen.bmj.com/site/about/guidelines.xhtml

above. Self-reported ethnicity was recorded under 16 categories and condensed to White, Mixed, Asian, Black/African/Caribbean, Chinese and Other. Categories of not asked and not stated were coded as missing. Smoking was self-reported as current smoking or non-smoking. Non-smokers were further asked if they had ever smoked or had previously smoked and quit. This was categorised as stopped more than 12 months before conception, stopped less than 12 months before conception or stopped when pregnancy confirmed. Employment was self-reported at booking appointment and categorised as employed, unemployed, in education, and not specified. Infertility treatment was categorised as no/investigations only and yes (hormonal only, in-vitro fertilisation, gamete intrafallopian transfer and other surgical) in either one or both pregnancies. Inter-pregnancy interval was defined as the interval between the first live birth and conception of the second pregnancy. The difference in days between two consecutive live births was calculated and gestational age of the latter birth subtracted from this to derive the inter-pregnancy interval.

Statistical analysis

All analysis was performed using Stata 15[29]. Univariable comparisons were carried out using ANOVA for continuous variables and chi square test for categorical variables. Linear regression was used to examine the association of maternal change in BMI between pregnancies in the sub-sample of women who gained ≥1 BMI unit weight (assessed as a continuous variable in kg/m^2) with birthweight (assessed as a continuous variable in grams).

Logistic regression was then used to examine the association between the categorised variable of maternal change in BMI with risk of LGA first in the whole sample and then stratified by maternal BMI category in first pregnancy. Risk of LGA was explored in the full sample adjusting for previous pregnancy outcome of LGA. The risk of LGA in second pregnancy after having a non-LGA baby in the first pregnancy was explored in a sub-sample of women who had non-LGA births in the first pregnancy. The risk of recurrent LGA (LGA in both pregnancies) was explored in a sub-sample of women who had LGA births in the first pregnancy.

Initial univariable analysis was followed by multivariable models adjusting for potential confounding factors – maternal age, ethnicity, highest educational qualification, whether or not undergone infertility treatment, employment status, smoking behaviour in second pregnancy, gestational diabetes in second pregnancy and inter-pregnancy interval. Linear regression models with birthweight as the outcome were also adjusted for gestational age at birth and birthweight in previous pregnancy.

A statistical significance level of 0.05 with 95% confidence intervals was used in the regression models.

Ethical considerations

All data were anonymised to the research team. Ethics approval was granted by the University of Southampton Faculty of Medicine Ethics Committee: study ID 25508.

Patient and Public Involvement

Patients and public were not involved in setting the research question or the outcome measures, nor were they involved in developing plans for the design or implementation of the study.

For peer review only - http://bmjopen.bmj.com/site/about/guidelines.xhtml

For peer review only

Results

The first and second pregnancies of 15940 women were included. Of these, 16.0% of women lost ≥1 BMI unit, 36.3% remained weight stable (-1 to 1 BMI unit) and 47.7% gained ≥1 BMI unit between their first and second live birth pregnancies. Weight loss of >2 BMI units was observed in 7.3% of women whereas 10.7% gained >2 BMI units and 19.8% gained three or more BMI units. Mean BMI at second pregnancy booking was 27.9 kg/m^2 (standard deviation (SD) 5.8) in women who gained weight (≥1 BMI Unit), 24.1 kg/m^2 (SD 5.1) in women who lost weight, and 23.8 kg/m^2 (SD 4.4) women whose weight remained stable between pregnancies (p<0.001) (Table 1).

Women who gained ≥1 BMI unit by the start of their second pregnancy were more likely to be smokers, unemployed, with lower educational attainment and to have a longer inter-pregnancy interval, compared to those who maintained a stable weight between pregnancies. Mean maternal age was lowest in the women who gained weight (28.4 years, standard deviation 5.5) and highest in the women who remained weight stable (29.8 years, standard deviation 5.3).

Within mothers who gained weight, 35.8% were in the normal weight BMI category, 34.3% in the overweight category, and 29.5% in the obese category by the start of their second pregnancy. This compares to 66.8%, 19.7% and 9.2% respectively within those with stable inter-pregnancy weight.

Birthweight (grams) was significantly higher in babies born to women who gained weight between pregnancies (3517g, SD 45) compared to those born to women who lost weight and remained weight stable where the mean birthweight was comparable (3463g, SD 563, 3467g, SD 523 respectively) (p<0.001). A lower proportion of babies born to women who lost weight (12.4%) or remained weight stable (11.9%) between pregnancies were LGA compared to 14.5% in women who gained weight (p<0.001). Compared to normal weight women, overweight and obese women were at increased risk of LGA births in both pregnancies with risk highest in obese women (unadjusted odds ratio 2.2, 95% CI 1.9 to 2.6, p<0.001 and unadjusted odds ratio 2.1, 95% CI 1.8 to 2.3, p<0.001 in first and second pregnancy respectively).

Figure 1 shows the percentage of LGA as recurrent LGA (first and second pregnancy) or LGA in second pregnancy only (after having a non-LGA baby in the first pregnancy) by the inter-pregnancy change in maternal BMI stratified by maternal BMI category in the first pregnancy. The lowest proportion of LGA births in the second pregnancy was in underweight women in the first pregnancy who remained weight stable (5.9%), while the highest was in obese women who gained weight (20.2%). Recurrent LGA was lowest in normal weight and overweight women who lost weight and highest in obese women who lost weight.

There was a significant positive association between birthweight in the second pregnancy with each unit increase in BMI between pregnancies. The largest increase was in normal weight women who gained ≥1 BMI unit in weight between pregnancies (adjusted increase in birthweight per unit increase in maternal BMI (13.0g, 95% CI 7.1 to 19.0, p<0.001) (Table 2).

The logistic regression models show an increased risk of LGA in the second pregnancy in the full sample on weight gain compared to remaining weight stable. When stratified by baseline BMI category, there was a significantly increased risk of LGA birth in the second pregnancy in normal weight women who gained ≥1 BMI unit weight (adjusted odds ratio (aOR) 1.31, 95% CI 1.13 to 1.52, p<0.001) compared to the reference group of normal-

For peer review only - http://bmjopen.bmj.com/site/about/guidelines.xhtml

weight women who remained weight stable (Table 3). No association was observed between the risk of LGA and maternal BMI change in underweight, overweight and obese women.

There was a significantly reduced risk of recurrent LGA birth in the second pregnancy in overweight women who had a LGA infant in the first pregnancy and lost ≥1 BMI unit in weight (aOR 0.44, 95% CI 0.23 to 0.85, p=0.02) (Table 4). No association was observed between risk of recurrent LGA and maternal BMI change in underweight, normal weight and obese women.

There was a significantly increased risk of 'new' LGA birth in the second pregnancy after having a non-LGA infant in the first pregnancy in normal weight and overweight women who had gained ≥1 BMI unit weight (aOR 1.39, 95% CI 1.18 to 1.63, p>0.001, aOR 1.31, 95% CI 1.03 to 1.66, p=0.03 respectively) (Table 5). No association was observed between the risk of new LGA in the second pregnancy and maternal BMI inter-pregnancy change in underweight and obese women.

Discussion

This study examined the association of change in women's BMI between their first and second live birth pregnancies with LGA risk in their second pregnancy in a population-based cohort of 15940 women in the South of England. A large proportion (48%) of women gained ≥1 BMI unit in weight when presenting to antenatal care for their second pregnancy. The proportion of LGA births in women who lost weight was 12.4% and 11.9% in those that remained weight stable compared to 14.5% in women who gained weight. Normal weight women who gained ≥1 BMI unit by the start of their second pregnancy had an increased risk of an LGA birth. Overweight women who lost ≥1 BMI unit were had a reduced risk of recurrent LGA, whereas both normal and overweight women who gained ≥1 BMI unit between pregnancies had an increased risk of LGA birth in their second pregnancy after a non-LGA birth in the first.

Compared to the population-based Swedish cohort which carried out a similar analysis for LGA and other outcomes in 151025 women, a lower proportion of women remained weight stable in this cohort (46% compared to 36%) and a higher proportion lost (11% compared to 16%) or gained (43% compared to 48%) weight. Amongst women who gained weight, a higher proportion gained 3 or more BMI units in this cohort (20%) compared to the Swedish cohort (11%)[23]. Similarly, in comparison to a population-based cohort of 24520 women in Aberdeen, Scotland; a larger proportion of women in this study lost (4.8% compared to 7.3%) or gained (25.6% compared to 30.5%) weight (>2 BMI units) [26]. The Swedish cohort used data from 1992 to 2001 and the Scottish cohort from 1986 to 2013. The differences could reflect the increase in the prevalence of maternal overweight and obesity over time.

We showed an increased risk of LGA in the second pregnancy in the full sample on weight gain compared to weight remaining stable. This effect remained after adjusting for previous outcome of LGA (in first pregnancy). On stratification by BMI, this effect was only observed in normal weight woman. In a population-based cohort in the US, women were found to be at increased risk of LGA in the second pregnancy if their pre-pregnancy BMI category changed towards overweight or obese from first to second pregnancy regardless of their BMI category in first pregnancy except in underweight women who increased to normal weight. This study only examined risk in second pregnancy without adjustment for outcome in first pregnancy and considered weight change as change in BMI category only[21].

In obese women in the US, inter-pregnancy weight gain of ≥2 BMI units was associated with increased risk of LGA and a weight loss of ≥2 BMI units was associated with decreased risk

For peer review only - http://bmjopen.bmj.com/site/about/guidelines.xhtml

compared to the reference group of weight maintained between 2 BMI units[22]. We found no association between weight change and risk of second pregnancy LGA in obese women although it may be that as obese women are already at increased risk of LGA births the change in BMI between-pregnancy in this cohort was not large enough to detect a further increase in risk.

Risk of recurrent LGA was analysed in one previous study in Scotland which found that inter-pregnancy weight gain (≥2 BMI units) was associated with increased risk of recurrent LGA and weight loss (≥2 BMI units) was found to be protective. Stratification by BMI showed that women with healthy weight (BMI <25kg/m^2) were at increased risk of recurrent LGA on gaining weight whereas overweight women (BMI ≥25kg/m^2) were at reduced risk of recurrent LGA on losing weight[26]. We showed a similar reduction in risk in overweight women who lost ≥1 BMI unit between pregnancies, but found no association in normal weight women.

We showed an increased risk of ‘new’ LGA in the second pregnancy (after a non-LGA birth in the first pregnancy) on weight gain compared to remaining weight stable. After stratification by BMI, we found that this association between inter-pregnancy weight gain and new LGA remained only in normal-weight and overweight women. The findings from this study are in line with findings with other studies in Scotland[24] and Sweden[23] which found increased risk of ‘new’ LGA with modest (1-3 BMI units) and large (≥3 BMI units) weight gain. Both studies also found a decreased risk with between pregnancy weight loss of >1 BMI unit which was not found in our study. Both studies stratified BMI as < and ≥25kg/m^2 and thus this is not directly comparable with our analysis as we further stratified the ≥25kg/m^2 category as overweight (BMI 25-29.9kg/m^2) and obese (≥30kg/m^2) and found an increased risk of new LGA in overweight, but not in obese women.

Women included in this analysis had a range of inter-pregnancy interval of less than 1 to up to 12 years and thus weight change could be due to postpartum weight retention or late postpartum weight gain. A study looking at the effects of pregnancy on long-term weight gain concluded that women who had not lost pregnancy weight at one year postpartum were more likely to retain weight longer term[30]. We examined the risk of maternal weight gain with length of the inter-pregnancy interval and found that women with an interval of 12-23 months were least likely to start the next pregnancy at a higher weight[31]. We also examined inter-pregnancy interval as a predictor for risk of LGA and found no association but found that an interval of 12-23 months was associated with lower risk of small-for-gestational age (SGA) (data not shown). In this study, we have adjusted for the length of the inter-pregnancy interval in the models.

Future research that characterises the predictors of postpartum weight change would help design interventions to support postpartum weight loss. Key to this is an understanding of the pattern of weight change during this period as well as identifying the optimal setting and delivery of the intervention. Advice regarding healthy eating and physical activity is more commonly received during pregnancy but when advice is received postpartum, it was found not to be associated with healthy diet or physical activity behaviours[32]. Most interventions that have been successful in limiting and promoting weight loss were combined diet and physical activity interventions with self-monitoring[33]. However, the timing of engaging women and length of intervention or engagement are important with one study showing that an intervention from 16 weeks pregnancy to six months postpartum was more effective than the same intervention from birth to six months postpartum intervention[34].

As pregnancy and early postpartum is a period of major change for women and their families, interventions need to be carefully designed to be attractive, flexible and feasible for women at this stage with competing priorities and time demands. Contact as part of these interventions also needs to be medium to long term to allow for weight maintenance. The majority of appointments during the postpartum period in the UK are child health and development reviews with the health visitor and one with the general practitioner in the first

For peer review only - http://bmjopen.bmj.com/site/about/guidelines.xhtml

two years after birth. The feasibility of using these appointments to engage and support maternal weight and health needs to be explored.

Strengths and limitations

This is a relatively large population-based cohort including women from various socioeconomic and ethnic backgrounds delivering at a large maternity centre in Southampton, UK, thus representative of the regional population. One city may not be representative of the national population, and according to the UK Department of Communities and Local Government English indices of deprivation report, Southampton is more deprived than average with the situation having worsened between 2010 and 2015[35]. However, about half of the women included in this analysis reside in the rest of Hampshire (the region where Southampton is situated), which is less deprived. The sample was 87% White comparable to the 2011 England and Wales population census of 86% White[36]. The analysis was adjusted for several key confounders that were reasonably complete (96% complete for ethnicity and employment status). Both the exposure and outcome in this study were objectively measured by healthcare professionals as part of routine antenatal and delivery care.

An important limitation was the lack of information on weight gain during pregnancy, which is a key factor influencing risk of LGA births[37]. Women who had their first booking appointment later into the pregnancy (more than 24 weeks) were excluded from the analysis in order to ensure comparability of weight measurements between pregnancies. Some of the confounding factors which were accounted for in the analysis were self-reported, however the information was collected prospectively, therefore any measurement error in likely to be non-differential. Another limitation is that these findings are based on observational data so inferences about causation cannot be drawn and the risk of residual confounding influencing the results needs to be considered. However, it is not feasible or ethical to conduct a randomised trial to address the aim of this study.

In conclusion, a large proportion of women gained weight between their first and second pregnancy, and a higher proportion of these women had a LGA birth in their second pregnancy compared to their first in this English cohort. Overall, weight gain between pregnancies was associated with an increased risk of LGA in the second pregnancy. Risk of new LGA was higher in normal weight and overweight women who gained weight after a non-LGA birth in their first pregnancy compared who remained weight stable. Overweight women who had a LGA birth in their first pregnancy were at a lower risk of a recurrent LGA birth in their second pregnancy if they lost weight between pregnancies. Supporting efforts to lose weight in overweight and obese women between pregnancies, and stop weight gain in all women planning to have further children (except those who are underweight) are important preventive measures of subsequent adverse maternal and offspring health outcomes.

Author Contributions

Study design (NZ, PJR, NSM, NAA), data analysis (NZ, SW), acquisition and interpretation of the data (NZ, NAA), drafting of the abstract (NZ), revising for content (NZ, SW, PJR, NSM, NAA) and approval of final version before submission (NZ, SW, PJR, NSM, NAA).

Data statement

Anonymised data are only available upon request from the authors conditional on the approval by the appropriate institutional ethics and research governance processes.

For peer review only - http://bmjopen.bmj.com/site/about/guidelines.xhtml

Acknowledgements

The authors would like to thank David Cable (Electronic Patient Records Implementation and Service Manager) at University Hospital Southampton for support in accessing the data used in this study. They would also like to thank their clinical colleagues at the Princess Anne Hospital Southampton for collecting the data and making it available for analysis.

Funding

This work is supported by a University of Southampton Primary Care and Population Sciences PhD studentship (to NZ) , the Academy of Medical Sciences and the Wellcome Trust (Grant no: AMS_HOP001\1060 to NAA). NAA is also in receipt of research support from the National Institute for Health Research through the NIHR Southampton Biomedical Research Centre.

Competing interests

The authors have no competing interests to declare.

References

1. Heslehurst N, Rankin J, Wilkinson JR, et al. A nationally representative study of maternal obesity in England, UK: trends in incidence and demographic inequalities in 619 323 births, 1989-2007. *Int J Obes (Lond)* 2010;34(3):420-8.
2. Poston L. Maternal obesity, gestational weight gain and diet as determinants of offspring long term health. *Best Pract Res Clin Endocrinol Metab* 2012;26(5):627-39.
3. Barker DJP. Fetal origins of coronary heart disease. *BMJ* 1995;311:171-74.
4. Kramer MS, Morin I, Yang H, et al. Why are babies getting bigger? Temporal trends in fetal growth and its determinants. *J Pediatr* 2002;141(4):538-42.
5. Surkan PJ, Hsieh CC, Johansson AL, et al. Reasons for increasing trends in large for gestational age births. *Obstet Gynecol* 2004;104(4):720-6.
6. Hunt KJ, Schuller KL. The Increasing Prevalence of Diabetes in Pregnancy. *Obstet Gynecol Clin North Am.* 2007;34(2):173-99.
7. Ignell C, Claesson R, Anderberg E, et al. Trends in the prevalence of gestational diabetes mellitus in southern Sweden, 2003-2012. *Acta Obstet Gynecol Scand* 2014;93(4):420-4.
8. Casey BM, Lucas MJ, McIntire DD, et al. Pregnancy Outcomes in Women With Gestational Diabetes Compared With the General Obstetric Population. *Obstetrics & Gynecology* 1997;90(6):869-73.
9. Reilly JJ, Armstrong J, Dorosty AR, et al. Early life risk factors for obesity in childhood: cohort study. *BMJ* 2005;330(7504):1357.
10. Danielzik S, Czerwinski-Mast M, Langnase K, et al. Parental overweight, socioeconomic status and high birth weight are the major determinants of overweight and obesity in 5-7 y-old children: baseline data of the Kiel Obesity Prevention Study (KOPS). *Int J Obes Relat Metab Disord* 2004;28(11):1494-502.
11. Curhan GC, Willett WC, Rimm EB, et al. Birth Weight and Adult Hypertension, Diabetes Mellitus, and Obesity in US Men. *Circulation* 1996;94:3246-50.
12. Curhan GC, Chertow GM, Willett WC, et al. Birth Weight and Adult Hypertension and Obesity in Women. *Circulation* 1996;94:1310-15.

For peer review only - http://bmjopen.bmj.com/site/about/guidelines.xhtml

13. Phillips DIW, Young JB. Birth weight, climate at birth and the risk of obesity in adult life. *International Journal of Obesity* 2000;24:281-87.
14. Baptiste-Roberts K, Nicholson WK, Wang NY, et al. Gestational diabetes and subsequent growth patterns of offspring: the National Collaborative Perinatal Project. *Matern Child Health J* 2012;16(1):125-32.
15. Zhu Y, Olsen SF, Mendola P, et al. Growth and obesity through the first 7 y of life in association with levels of maternal glycemia during pregnancy: a prospective cohort study. *Am J Clin Nutr* 2016;103(3):794-800.
16. Marchi J, Berg M, Dencker A, et al. Risks associated with obesity in pregnancy, for the mother and baby: a systematic review of reviews. *Obes Rev* 2015;16(8):621-38.
17. Swamy GK, Edwards S, Gelfand A, et al. Maternal age, birth order, and race: differential effects on birthweight. *J Epidemiol Community Health* 2012;66(2):136-42.
18. Wilcox MA, Chang AMZ, Johnson IR. The effects of parity on birthweight using successive pregnancies. *Acta Obstetricia et Gynecologica Scandinavica* 1996;75(5):459-63.
19. Beaty TH, Skjaerven R, Breazeale DR, et al. Analyzing Sibship Correlations in Birth Weight Using Large Sibships From Norway. *Genetic Epidemiology* 1997;14:423-33.
20. Hinkle SN, Albert PS, Mendola P, et al. The association between parity and birthweight in a longitudinal consecutive pregnancy cohort. *Paediatr Perinat Epidemiol* 2014;28(2):106-15.
21. Getahun D, Ananth CV, Peltier MR, et al. Changes in prepregnancy body mass index between the first and second pregnancies and risk of large-for-gestational-age birth. *Am J Obstet Gynecol* 2007;196(6):530 e1-8.
22. Jain AP, Gavard JA, Rice JJ, et al. The impact of interpregnancy weight change on birthweight in obese women. *Am J Obstet Gynecol* 2013;208(3):205 e1-7.
23. Villamor E, Cnattingius S. Interpregnancy weight change and risk of adverse pregnancy outcomes: a population-based study. *Lancet* 2006;368(9542):1164-70.
24. Wallace JL, Bhattacharya S, Campbell DM, et al. Inter-pregnancy weight change impacts placental weight and is associated with the risk of adverse pregnancy outcomes in the second pregnancy. *BMC Pregnancy and Childbirth* 2014;14(40).
25. McBain RD, Dekker GA, Clifton VL, et al. Impact of inter-pregnancy BMI change on perinatal outcomes: a retrospective cohort study. *Eur J Obstet Gynecol Reprod Biol* 2016;205:98-104.
26. Wallace JM, Bhattacharya S, Campbell DM, et al. Inter-Pregnancy Weight Change and the Risk of Recurrent Pregnancy Complications. *PLoS One* 2016;11(5):e0154812.
27. National Institute for Health Care and Excellence. Antenatal care for uncomplicated pregnancies. Manchester, UK, 2008.
28. Norris T, Seaton SE, Manktelow BN, et al. Updated birth weight centiles for England and Wales. *Archives of Disease in Childhood. Fetal and Neonatal Edition* 2017.
29. Stata Statistical Software: Release 15 [program]. College Station, TX: StataCorp LLC, 2017.
30. Linné Y, Dye L, Barkeling B, et al. Long-Term Weight Development in Women: A 15-Year Follow-up of the Effects of Pregnancy. *Obesity Research* 2004;12(7):1166-78.
31. Ziauddeen N, Roderick PJ, Macklon NS, et al. Length of inter-pregnancy interval and subsequent preconception adiposity: Findings from a population-based cohort in the South of England. *Revue d'Épidémiologie et de Santé Publique* 2018;66:S374.
32. van der Pligt P, Olander EK, Ball K, et al. Maternal dietary intake and physical activity habits during the postpartum period: associations with clinician advice in a sample of Australian first time mothers. *BMC Pregnancy Childbirth* 2016;16:27.
33. van der Pligt P, Willcox J, Hesketh KD, et al. Systematic review of lifestyle interventions to limit postpartum weight retention: implications for future opportunities to prevent maternal overweight and obesity following childbirth. *Obes Rev* 2013;14(10):792-805.
34. Huang TT, Yeh CY, Tsai YC. A diet and physical activity intervention for preventing weight retention among Taiwanese childbearing women: a randomised controlled trial. *Midwifery* 2011;27(2):257-64.

For peer review only - http://bmjopen.bmj.com/site/about/guidelines.xhtml

35. Department for Communities and Local Government. The English Indices of Deprivation 2015, 2015.
36. Office for National Statistics. Ethnicity and National Identity in England and Wales 201, 2012.
37. Nohr EA, Vaeth M, Baker JL, et al. Combined associations of prepregnancy body mass index and gestational weight gain with the outcome of pregnancy. *American Journal of Clinical Nutrition* 2008;87(6):1750-59.

Figure legend

Figure 1: The percentage of LGA births in second pregnancy stratified by maternal BMI category and previous outcome of LGA

Table legends:

Table 1: Maternal and birth characteristics in second live birth pregnancy categorised by weight loss/no change and weight gain from previous pregnancy for the period of January 2003 - September 2017, University Hospital Southampton NHS Foundation Trust, Southampton, Hampshire, England

Table 2: Linear regression estimates for the association between birthweight (in g) in second live birth pregnancy with inter-pregnancy change in maternal BMI in the sub-sample of women who gained >= 1 BMI unit stratified by maternal body mass index (BMI) category at the first pregnancy

Table 3: Logistic regression models testing the association between risk of LGA in the second pregnancy and change in maternal body mass index (BMI) measured at the first antenatal visit of each pregnancy stratified by BMI category at the start of first pregnancy

Table 4: Logistic regression models testing the association between risk of recurrent LGA in the second pregnancy and change in maternal body mass index (BMI) measured at the first antenatal visit of each pregnancy stratified by BMI category in first pregnancy

Table 5: Logistic regression models testing the association between the risk of LGA birth in the second pregnancy following a non-LGA birth in the first pregnancy and change in maternal body mass index (BMI) measured at the first antenatal visit of each pregnancy stratified by BMI category in first pregnancy

For peer review only

For peer review only - http://bmjopen.bmj.com/site/about/guidelines.xhtml

Table 1: Maternal and birth characteristics in second live birth pregnancy categorised by weight loss/no change and weight gain from previous pregnancy for the period of January 2003 - September 2017, University Hospital Southampton NHS Foundation Trust, Southampton, Hampshire, England

	Lost <= -1 BMI units from previous pregnancy	Weight stable (>-1 to <1 BMI unit)	Gained >=1 BMI units from previous pregnancy	p
N	2548	5785	7607	
Maternal age, years (mean ± SD)	28.7 ± 5.4	29.8 ± 5.3	28.4 ± 5.5	<0.001
Timing of first booking appointment, weeks (mean ± SD)	10.8 ± 2.3	11.0 ± 2.3	11.1 ± 2.5	<0.001
Maternal BMI at booking, kg/m^2 (mean ± SD)	24.1 ± 5.1	23.8 ± 4.4	27.9 ± 5.8	<0.001
Maternal BMI at booking (%, 95% CI)				
Underweight (< 18.5)	6.9 (5.9 to 7.9)	4.3 (3.8 to 4.8)	0.4 (0.3 to 0.6)	<0.001
Normal weight (18.5 to 24.9)	61.1 (59.2 to 63.0)	66.8 (65.6 to 68.1)	35.8 (34.7 to 36.9)	
Overweight (25.0 to 29.9)	20.1 (18.6 to 21.7)	19.7 (18.7 to 20.7)	34.3 (33.2 to 35.4)	
Obese (≥30.0)	11.9 (10.7 to 13.3)	9.2 (8.5 to 10.0)	29.5 (28.5 to 30.5)	
Maternal smoking status at booking (%, 95% CI)				
Never smoked/quit	57.2 (55.3 to 59.2)	63.0 (61.8 to 64.3)	56.4 (55.3 to 57.6)	<0.001
Stopped >1 year before conceiving	16.1 (14.6 to 17.5)	17.2 (16.3 to 18.2)	16.5 (15.7 to 17.4)	
Stopped <1 year prior to conceiving	4.0 (3.3 to 4.8)	2.8 (2.4 to 3.2)	4.1 (3.7 to 4.6)	
Stopped when pregnancy confirmed	6.8 (5.8 to 7.8)	5.9 (5.3 to 6.6)	8.3 (7.7 to 9.0)	
Continued smoking	15.9 (14.5 to 17.4)	11.0 (10.2 to 11.8)	14.6 (13.8 to 15.4)	
Maternal education (%, 95% CI)				
Secondary (GCSE) or under	30.7 (28.9 to 32.5)	24.0 (22.9 to 25.2)	32.3 (31.2 to 33.3)	<0.001
College (A levels)	40.4 (38.5 to 42.3)	38.8 (37.6 to 40.1)	42.1 (40.1 to 43.2)	
University degree or above	28.9 (27.2 to 30.7)	37.1 (35.9 to 38.4)	25.6 (24.6 to 26.6)	
Maternal employment (%, 95% CI)				
Employed	66.2 (64.3 to 68.0)	71.7 (70.5 to 72.9)	62.7 (61.6 to 63.8)	<0.001
Unemployed	31.8 (30.0 to 33.7)	26.9 (25.8 to 28.1)	35.4 (34.4 to 36.5)	
In education	0.9 (0.6 to 1.4)	0.8 (0.6 to 1.1)	1.2 (0.9 to 1.4)	
Not specified	1.0 (0.7 to 1.5)	0.6 (0.4 to 0.8)	0.7 (0.5 to 0.9)	
Ethnicity (%, 95% CI)				
White	89.9 (88.7 to 91.1)	88.0 (87.1 to 88.8)	85.0 (84.2 to 85.8)	<0.001
Mixed	0.8 (0.5 to 1.3)	0.9 (0.7 to 1.2)	1.5 (1.2 to 1.8)	
Asian	4.2 (3.5 to 5.1)	5.0 (4.4 to 5.6)	6.9 (6.4 to 7.5)	

For peer review only

Black/African/Caribbean	0.6 (0.4 to 1.0)	1.0 (0.8 to 1.3)	1.9 (1.6 to 2.3)	
Chinese	0.5 (0.3 to 0.9)	0.7 (0.5 to 0.9)	0.5 (0.3 to 0.7)	
Other	0.7 (0.4 to 1.1)	1.0 (0.8 to 1.3)	1.1 (0.9 to 1.4)	
Not specified	3.1 (2.5 to 3.9)	3.5 (3.0 to 4.0)	3.0 (2.7 to 3.5)	
Inter-pregnancy interval (median, IQR)	21.7 (14.4 to 32.7)	21.6 (14.1 to 32.0)	25.0 (15.0 to 39.7)	<0.001
Inter-pregnancy interval (%, 95% CI)				
0-11 months	17.4 (15.9 to 18.9)	17.6 (16.6 to 18.6)	17.5 (16.7 to 18.4)	<0.001
12-23 months	39.8 (37.8 to 41.7)	39.9 (38.6 to 41.1)	30.3 (29.3 to 31.3)	
24-35 months	22.6 (21.0 to 24.2)	23.6 (22.5 to 24.7)	22.8 (21.9 to 23.8)	
36 months or more	20.3 (18.7 to 21.9)	18.9 (17.9 to 20.0)	29.4 (28.4 to 30.4)	
Birthweight, grams (mean ± SD)	3463 ± 563	3467 ± 523	3517 ± 545	<0.001
Previous size at birth (first pregnancy)				
Small-for-gestational age	13.1 (11.8 to 14.4)	12.6 (11.8 to 13.5)	12.0 (11.3 to 12.8)	0.17
Appropriate-for-gestational age	79.6 (77.9 to 81.1)	81.1 (80.0 to 82.1)	80.7 (79.8 to 81.6)	
Large-for-gestational age	7.4 (6.4 to 8.5)	6.3 (5.7 to 7.0)	7.3 (6.7 to 7.9)	
Size at birth (second pregnancy)				
Small-for-gestational age	8.7 (7.6 to 9.8)	7.0 (6.4 to 7.7)	6.4 (5.9 to 7.0)	<0.001
Appropriate-for-gestational age	79.0 (77.3 to 80.5)	81.1 (80.0 to 82.1)	79.1 (78.2 to 80.0)	
Large-for-gestational age	12.4 (11.1 to 13.7)	11.9 (11.1 to 12.8)	14.5 (13.7 to 15.3)	

For peer review only - http://bmjopen.bmj.com/site/about/guidelines.xhtml

Table 2: Linear regression estimates for the association between birthweight (in g) in second live birth pregnancy with inter-pregnancy change in maternal BMI in the sub-sample of women who gained >= 1 BMI unit stratified by maternal body mass index (BMI) category at the first pregnancy

	First to second pregnancy														
	Full sample			Underweight at first pregnancy			Normal weight at first pregnancy			Overweight at first pregnancy			Obese at first pregnancy		
	n	Birthweight (g) per unit increase in maternal BMI change (95% CI)	p	n	Birthweight (g) per unit increase in maternal BMI change (95% CI)	p	n	Birthweight (g) per unit increase in maternal BMI change (95% CI)	p	n	Birthweight (g) per unit increase in maternal BMI change (95% CI)	p	n	Birthweight (g) per unit increase in maternal BMI change (95% CI)	p
Model 1	7607	8.3<br>3.8 to 12.9	<0.001	353	1.5<br>-20.4 to 23.3	0.89	4326	10.0<br>3.6 to 16.5	0.002	1913	-3.8<br>-12.0 to 4.5	0.37	1015	2.3<br>-10.0 to 14.6	0.72
Model 2	7607	6.8<br>2.8 to 10.7	0.001	353	-4.0<br>-23.0 to 15.0	0.68	4326	9.3<br>3.7 to 14.9	0.001	1913	-1.6<br>-8.8 to 5.7	0.67	1015	2.9<br>-8.0 to 13.7	0.60
Model 3	7324	9.9<br>5.9 to 14.0	<0.001	338	-2.4<br>-21.5 to 16.8	0.81	4154	12.0<br>6.2 to 17.7	<0.001	1839	4.3<br>-3.3 to 11.8	0.27	993	4.6<br>-6.7 to 15.8	0.43
Model 4	7324	7.8<br>3.7 to 11.9	<0.001	338	-3.0<br>-22.3 to 16.4	0.76	4154	12.1<br>6.3 to 17.9	<0.001	1839	4.8<br>-2.7 to 12.4	0.21	993	4.3<br>-6.9 to 15.6	0.45
Model 5	7324	9.0<br>4.8 to 13.2	<0.001	338	-2.8<br>-22.3 to 16.6	0.78	4154	13.0<br>7.1 to 19.0	<0.001	1839	6.1<br>-1.7 to 13.8	0.12	993	6.6<br>-5.0 to 18.1	0.26

Model 1 is adjusted for: gestational age at birth
Model 2 is adjusted for: gestational age at birth, birth weight (previous pregnancy) and gestational age at birth (previous pregnancy)
Model 3 is adjusted for: gestational age at birth, birth weight (previous pregnancy) and gestational age at birth (previous pregnancy), maternal age, ethnicity, highest educational qualification, whether undergone infertility treatment, smoking status, employment status and infant gender
Model 4 is adjusted for: gestational age at birth, birth weight (previous pregnancy) and gestational age at birth (previous pregnancy), maternal age, ethnicity, highest educational qualification, whether undergone infertility treatment, smoking status, employment status, infant gender, baseline BMI and gestational diabetes in current pregnancy
Model 5 is adjusted for: gestational age at birth, birth weight (previous pregnancy) and gestational age at birth (previous pregnancy), maternal age, ethnicity, highest educational qualification, whether undergone infertility treatment, smoking status, employment status, infant gender, baseline BMI, gestational diabetes in current pregnancy and inter-pregnancy interval

For peer review only - http://bmjopen.bmj.com/site/about/guidelines.xhtml

Table 3: Logistic regression models testing the association between risk of LGA in the second pregnancy and change in maternal body mass index (BMI) measured at the first antenatal visit of each pregnancy stratified by BMI category at the start of first pregnancy

	Maternal BMI change (categorised)	First to second pregnancy														
		Full sample			Underweight at first pregnancy			Normal weight at first pregnancy			Overweight at first pregnancy			Obese at first pregnancy		
		n	Odds ratio, OR (95% CI)	p	n	Odds ratio, OR (95% CI)	p	n	Odds ratio, OR (95% CI)	p	n	Odds ratio, OR (95% CI)	p	n	Odds ratio, OR (95% CI)	p
Unadjusted	Lost <= -1 BMI units from previous pregnancy	15922	1.04<br>0.90 to 1.20	0.56	599	-	-	9427	0.79<br>0.63 to 0.99	0.04	3800	0.86<br>0.67 to 1.12	0.27	2075	1.03<br>0.75 to 1.40	0.87
	Gained >=1 BMI units from previous pregnancy		1.25<br>1.13 to 1.39	<0.001		2.19<br>0.92 to 5.24	0.08		1.23<br>1.07 to 1.41	0.003		1.11<br>0.91 to 1.35	0.32		1.11<br>0.84 to 1.45	0.46
Model 1	Lost <= -1 BMI units from previous pregnancy	15897	1.01<br>0.87 to 1.17	0.90	599	-	-	9409	0.84<br>0.67 to 1.06	0.14	3796	0.83<br>0.63 to 1.09	0.17	2072	0.96<br>0.69 to 1.32	0.79
	Gained >=1 BMI units from previous pregnancy		1.23<br>1.11 to 1.37	<0.001		2.09<br>0.87 to 5.04	0.10		1.22<br>1.06 to 1.40	0.006		1.11<br>0.90 to 1.37	0.31		1.07<br>0.81 to 1.42	0.62
Model 2	Lost <= -1 BMI units from previous pregnancy	15281	1.04<br>0.89 to 1.21	0.64	553	-	-	9013	0.89<br>0.70 to 1.13	0.33	3650	0.85<br>0.64 to 1.12	0.25	2024	0.95<br>0.68 to 1.32	0.75
	Gained >=1 BMI units from previous		1.29<br>1.16 to 1.45	<0.001		1.65<br>0.65 to 4.20	0.29		1.30<br>1.12 to 1.51	<0.001		1.17<br>0.94 to 1.44	0.16		1.09<br>0.82 to 1.45	0.56

For peer review only

For peer review only - http://bmjopen.bmj.com/site/about/guidelines.xhtml

	pregnancy															
Model 3	Lost <= -1 BMI units from previous pregnancy	15281	0.88 0.76 to 1.03	0.13	544	-	-	9013	0.84 0.66 to 1.07	0.16	3650	0.84 0.64 to 1.11	0.23	2024	0.92 0.66 to 1.28	0.61
	Gained >=1 BMI units from previous pregnancy		1.23 1.10 to 1.37	<0.001		1.67 0.66 to 4.26	0.28		1.29 1.11 to 1.49	0.001		1.16 0.94 to 1.44	0.17		1.08 0.81 to 1.44	0.61
Model 4	Lost <= -1 BMI units from previous pregnancy	15281	0.89 0.76 to 1.04	0.14	544	-	-	9013	0.84 0.66 to 1.07	0.16	3650	0.84 0.64 to 1.12	0.24	2024	0.95 0.68 to 1.33	0.77
	Gained >=1 BMI units from previous pregnancy		1.24 1.11 to 1.39	<0.001		1.62 0.63 to 4.20	0.32		1.29 1.11 to 1.50	0.001		1.17 0.94 to 1.45	0.16		1.13 0.84 to 1.51	0.42

* Weight stable (>-1 to <1 BMI unit) was used as the reference group.

Model 1 is adjusted for: previous outcome of LGA
Model 2 is adjusted for: previous outcome of LGA, maternal age, ethnicity, highest educational qualification, whether undergone infertility treatment, smoking status, employment status and infant gender
Model 3 is adjusted for: previous outcome of LGA, maternal age, ethnicity, highest educational qualification, whether undergone infertility treatment, smoking status, employment status, infant gender, baseline BMI and gestational diabetes in current pregnancy
Model 4 is adjusted for: previous outcome of LGA, maternal age, ethnicity, highest educational qualification, whether undergone infertility treatment, smoking status, employment status, infant gender, baseline BMI, gestational diabetes in current pregnancy and inter-pregnancy interval

For peer review only

Table 4: Logistic regression models testing the association between risk of recurrent LGA in the second pregnancy and change in maternal body mass index (BMI) measured at the first antenatal visit of each pregnancy stratified by BMI category in first pregnancy

	Maternal BMI change (categorised)	First to second pregnancy											
		Full sample			Normal weight at first pregnancy			Overweight at first pregnancy			Obese at first pregnancy		
		n	Odds ratio, OR (95% CI)	p	n	Odds ratio, OR (95% CI)	p	n	Odds ratio, OR (95% CI)	p	n	Odds ratio, OR (95% CI)	P
Unadjusted	Lost <= -1 BMI units from previous pregnancy	1109	0.80 0.56 to 1.14	0.23	521	0.68 0.35 to 1.32	0.25	338	0.47 0.25 to 0.87	0.02	236	1.33 0.64 to 2.75	0.44
	Gained >=1 BMI units from previous pregnancy		0.93 0.72 to 1.21	0.60		0.89 0.62 to 1.28	0.54		0.67 0.41 to 1.12	0.13		1.45 0.75 to 2.80	0.27
Model 1	Lost <= -1 BMI units from previous pregnancy	1066	0.85 0.59 to 1.23	0.38	500	0.67 0.34 to 1.35	0.26	324	0.45 0.23 to 0.86	0.02	229	1.42 0.66 to 3.05	0.37
	Gained >=1 BMI units from previous pregnancy		1.01 0.77 to 1.34	0.94		0.99 0.67 to 1.46	0.95		0.71 0.42 to 1.21	0.21		1.50 0.75 to 3.00	0.25
Model 2	Lost <= -1 BMI units from previous pregnancy	1066	0.72 0.49 to 1.06	0.09	500	0.63 0.32 to 1.28	0.20	324	0.44 0.23 to 0.85	0.02	229	1.34 0.62 to 2.89	0.46
	Gained >=1 BMI units from previous pregnancy		0.97 0.73 to 1.29	0.84		0.97 0.65 to 1.43	0.86		0.70 0.41 to 1.20	0.20		1.51 0.75 to 3.01	0.25
Model 3	Lost <= -1 BMI units from previous pregnancy	1066	0.72 0.49 to 1.06	0.09	500	0.63 0.31 to 1.27	0.20	324	0.44 0.23 to 0.85	0.02	229	1.39 0.64 to 3.02	0.41
	Gained >=1 BMI units from previous pregnancy		0.97 0.73 to 1.29	0.86		0.96 0.64 to 1.42	0.83		0.70 0.41 to 1.20	0.20		1.59 0.79 to 3.22	0.19

For peer review only - http://bmjopen.bmj.com/site/about/guidelines.xhtml

* Weight stable (>-1 to <1 BMI unit) was used as the reference group.

Model 1 is adjusted for: maternal age, ethnicity, highest educational qualification, whether undergone infertility treatment, smoking status, employment status and infant gender

Model 2 is adjusted for: maternal age, ethnicity, highest educational qualification, whether undergone infertility treatment, smoking status, employment status, infant gender, baseline BMI and gestational diabetes in current pregnancy

Model 3 is adjusted for: maternal age, ethnicity, highest educational qualification, whether undergone infertility treatment, smoking status, employment status, infant gender, baseline BMI, gestational diabetes in current pregnancy and inter-pregnancy interval

For peer review only

Table 5: Logistic regression models testing the association between the risk of LGA birth in the second pregnancy following a non-LGA birth in the first pregnancy and change in maternal body mass index (BMI) measured at the first antenatal visit of each pregnancy stratified by BMI category in first pregnancy

	Maternal BMI change (categorised)	First to second pregnancy														
		Full sample			Underweight at first pregnancy			Normal weight at first pregnancy			Overweight at first pregnancy			Obese at first pregnancy		
		n	Odds ratio, OR (95% CI)	p	n	Odds ratio, OR (95% CI)	p	n	Odds ratio, OR (95% CI)	p	n	Odds ratio, OR (95% CI)	p	n	Odds ratio, OR (95% CI)	p
Unadjusted	Lost <= -1 BMI units from previous pregnancy	14788	1.06 0.90 to 1.24	0.51	585	-	-	8888	0.87 0.68 to 1.12	0.29	3458	0.95 0.70 to 1.28	0.72	1836	0.89 0.62 to 1.27	0.52
	Gained >=1 BMI units from previous pregnancy		1.30 1.16 to 1.46	<0.001		1.78 0.73 to 4.35	0.21		1.29 1.10 to 1.50	0.001		1.24 0.98 to 1.56	0.07		1.01 0.74 to 1.37	0.97
Model 1	Lost <= -1 BMI units from previous pregnancy	14215	1.08 0.92 to 1.28	0.34	540	-	-	8513	0.92 0.71 to 1.19	0.53	3326	0.98 0.72 to 1.33	0.89	1795	0.87 0.60 to 1.25	0.44
	Gained >=1 BMI units from previous pregnancy		1.36 1.21 to 1.54	<0.001		1.41 0.54 to 3.67	0.48		1.38 1.17 to 1.62	<0.001		1.30 1.02 to 1.65	0.03		1.02 0.75 to 1.40	0.89
Model 2	Lost <= -1 BMI units from previous pregnancy	14215	0.93 0.78 to 1.10	0.40	531	-	-	8513	0.87 0.67 to 1.13	0.30	3326	0.97 0.71 to 1.32	0.84	1795	0.84 0.58 to 1.21	0.35
	Gained >=1 BMI units from previous pregnancy		1.29 1.14 to 1.45	<0.001		1.43 0.55 to 3.71	0.47		1.36 1.16 to 1.60	<0.001		1.29 1.02 to 1.64	0.04		1.00 0.73 to 1.38	0.98
Model 3	Lost <= -1 BMI units from previous pregnancy	14215	0.93 0.79 to 1.11	0.43	531	-	-	8513	0.87 0.67 to 1.13	0.30	3326	0.97 0.71 to 1.32	0.86	1795	0.87 0.60 to 1.26	0.47
	Gained >=1		1.30	<0.001		1.39	0.51		1.37	<0.001		1.30	0.03		1.05	0.77

For peer review only

For peer review only - http://bmjopen.bmj.com/site/about/guidelines.xhtml

	BMI units from previous pregnancy		1.15 to 1.47			0.53 to 3.68			1.16 to 1.61			1.02 to 1.65			0.76 to 1.44	

* Weight stable (>-1 to <1 BMI unit) was used as the reference group.

Model 1 is adjusted for: maternal age, ethnicity, highest educational qualification, whether undergone infertility treatment, smoking status, employment status and infant gender
Model 2 is adjusted for: maternal age, ethnicity, highest educational qualification, whether undergone infertility treatment, smoking status, employment status, infant gender, baseline BMI and gestational diabetes in current pregnancy
Model 3 is adjusted for: maternal age, ethnicity, highest educational qualification, whether undergone infertility treatment, smoking status, employment status, infant gender, baseline BMI, gestational diabetes in current pregnancy and inter-pregnancy interval

For peer review only

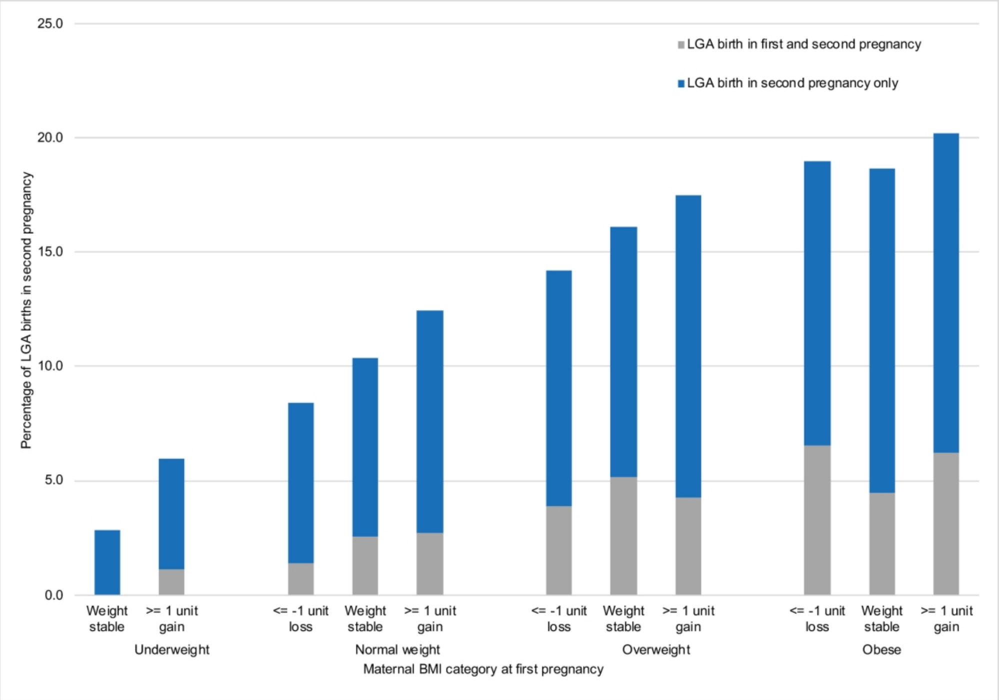

Figure 1: The percentage of LGA births in second pregnancy stratified by maternal BMI category and previous outcome of LGA

203x142mm (300 x 300 DPI)

Reporting checklist for cohort study.

Based on the STROBE cohort guidelines.

Instructions to authors

Complete this checklist by entering the page numbers from your manuscript where readers will find each of the items listed below.

Your article may not currently address all the items on the checklist. Please modify your text to include the missing information. If you are certain that an item does not apply, please write "n/a" and provide a short explanation.

Upload your completed checklist as an extra file when you submit to a journal.

In your methods section, say that you used the STROBE cohort reporting guidelines, and cite them as:

von Elm E, Altman DG, Egger M, Pocock SJ, Gotzsche PC, Vandenbroucke JP. The Strengthening the Reporting of Observational Studies in Epidemiology (STROBE) Statement: guidelines for reporting observational studies.

		Reporting Item	Page Number
Title	#1a	Indicate the study's design with a commonly used term in the title or the abstract	1 and 2
Abstract	#1b	Provide in the abstract an informative and balanced summary of what was done and what was found	2
Background / rationale	#2	Explain the scientific background and rationale for the investigation being reported	3-4
Objectives	#3	State specific objectives, including any prespecified hypotheses	4
Study design	#4	Present key elements of study design early in the paper	4
Setting	#5	Describe the setting, locations, and relevant dates, including periods of recruitment, exposure, follow-up, and data collection	4
Eligibility criteria	#6a	Give the eligibility criteria, and the sources and methods of selection of participants. Describe methods of follow-up.	4

For peer review only

	#6b	For matched studies, give matching criteria and number of exposed and unexposed	n/a
Variables	#7	Clearly define all outcomes, exposures, predictors, potential confounders, and effect modifiers. Give diagnostic criteria, if applicable	4-5
Data sources / measurement	#8	For each variable of interest give sources of data and details of methods of assessment (measurement). Describe comparability of assessment methods if there is more than one group. Give information separately for for exposed and unexposed groups if applicable.	4-5
Bias	#9	Describe any efforts to address potential sources of bias	n/a
Study size	#10	Explain how the study size was arrived at	4
Quantitative variables	#11	Explain how quantitative variables were handled in the analyses. If applicable, describe which groupings were chosen, and why	4-5
Statistical methods	#12a	Describe all statistical methods, including those used to control for confounding	5
	#12b	Describe any methods used to examine subgroups and interactions	5
	#12c	Explain how missing data were addressed	n/a
	#12d	If applicable, explain how loss to follow-up was addressed	n/a
	#12e	Describe any sensitivity analyses	n/a
Participants	#13a	Report numbers of individuals at each stage of study—eg numbers potentially eligible, examined for eligibility, confirmed eligible, included in the study, completing follow-up, and analysed. Give information separately for for exposed and unexposed groups if applicable.	5
	#13b	Give reasons for non-participation at each stage	n/a
	#13c	Consider use of a flow diagram	n/a
Descriptive data	#14a	Give characteristics of study participants (eg demographic, clinical, social) and information on exposures and potential	5-6

For peer review only

		confounders. Give information separately for exposed and unexposed groups if applicable.	
	#14b	Indicate number of participants with missing data for each variable of interest	13-14
	#14c	Summarise follow-up time (eg, average and total amount)	n/a
Outcome data	#15	Report numbers of outcome events or summary measures over time. Give information separately for exposed and unexposed groups if applicable.	5-6, 13-14
Main results	#16a	Give unadjusted estimates and, if applicable, confounder-adjusted estimates and their precision (eg, 95% confidence interval). Make clear which confounders were adjusted for and why they were included	6-7, 15-21
	#16b	Report category boundaries when continuous variables were categorized	4-5, 13-14
	#16c	If relevant, consider translating estimates of relative risk into absolute risk for a meaningful time period	n/a
Other analyses	#17	Report other analyses done—e.g., analyses of subgroups and interactions, and sensitivity analyses	n/a
Key results	#18	Summarise key results with reference to study objectives	7
Limitations	#19	Discuss limitations of the study, taking into account sources of potential bias or imprecision. Discuss both direction and magnitude of any potential bias.	8-9
Interpretation	#20	Give a cautious overall interpretation considering objectives, limitations, multiplicity of analyses, results from similar studies, and other relevant evidence.	9
Generalisability	#21	Discuss the generalisability (external validity) of the study results	8-9
Funding	#22	Give the source of funding and the role of the funders for the present study and, if applicable, for the original study on which the present article is based	10

The STROBE checklist is distributed under the terms of the Creative Commons Attribution License CC-BY. This checklist was completed on 22. August 2018 using http://www.goodreports.org/, a tool made by the EQUATOR Network in collaboration with Penelope.ai

For peer review only

BMJ Open

Is maternal weight gain between pregnancies associated with risk of large-for-gestational age birth? Analysis of a UK population-based cohort

Journal:	*BMJ Open*
Manuscript ID	bmjopen-2018-026220.R1
Article Type:	Research
Date Submitted by the Author:	03-Dec-2018
Complete List of Authors:	Ziauddeen, Nida; University of Southampton, Academic unit of Primary Care and Population Sciences, Faculty of Medicine<br>Wilding, Sam; University of Southampton, Academic unit of Primary Care and Population Sciences, Faculty of Medicine<br>Roderick, Paul; University of Southampton, Academic unit of Primary Care and Population Sciences, Faculty of Medicine<br>Macklon, Nicholas; University of Copenhagen, Zealand University Hospital, Department of Obstetrics and Gynaecology; London Women's Clinic<br>Alwan, Nisreen; University of Southampton, Academic unit of Primary Care and Population Sciences, Faculty of Medicine; University of Southampton and University Hospital Southampton NHS Foundation Trust, NIHR Southampton Biomedical Research Centre
**Primary Subject Heading**:	Epidemiology
Secondary Subject Heading:	Epidemiology, Public health
Keywords:	obesity, pregnancy, pregnancy outcome, weight gain, large-for-gestational age

SCHOLARONE™
Manuscripts

Is maternal weight gain between pregnancies associated with risk of large-for-gestational age birth? Analysis of a UK population-based cohort

Nida Ziauddeen[1], Sam Wilding[1], Paul J. Roderick[1], Nicholas S. Macklon[2,3], Nisreen A. Alwan[1,4]

[1] Academic Unit of Primary Care and Population Sciences, Faculty of Medicine, University of Southampton, Southampton, UK

[2] Department of Obstetrics and Gynaecology, University of Copenhagen, Zealand University Hospital, Roskilde 4000, Denmark

[3] London Women's Clinic, 113-115 Harley Street, London W1G 6AP

[4] NIHR Southampton Biomedical Research Centre, University of Southampton and University Hospital Southampton NHS Foundation Trust, Southampton SO16 6YD, UK

Author emails: N.Ziauddeen@soton.ac.uk, S.A.Wilding@soton.ac.uk, pjr@soton.ac.uk, nism@regionsjaelland.dk, N.A.Alwan@soton.ac.uk

Corresponding author: Nida Ziauddeen

Academic Unit of Primary Care and Population Sciences, Faculty of Medicine, Mailpoint 805, Level C (AC22), South Academic Block, Southampton General Hospital, Tremona Road, Southampton SO16 6YD, UK

Email: N.Ziauddeen@soton.ac.uk

Telephone number: 023 81 206287

Keywords: obesity, pregnancy, pregnancy outcome, weight gain, birth weight

Word count: 4206 words

For peer review only

Abstract

Objective: Maternal overweight and obesity during pregnancy increases the risk of large-for-gestational age (LGA) birth and childhood obesity. We aimed to investigate the association between maternal weight change between subsequent pregnancies and risk of having a LGA birth.

Design: Population-based cohort.

Setting: Routinely collected antenatal healthcare data between January 2003 and September 2017 at University Hospital Southampton, England.

Participants: Health records of women with their first two consecutive singleton live-birth pregnancies were analysed (n=15940).

Primary outcome measure: Risk of LGA, recurrent LGA and 'new' LGA births in the second pregnancy.

Results: Of the 15940 women included, 16.0% lost and 47.7% gained weight (≥1 kg/m^2) between pregnancies. A lower proportion of babies born to women who lost ≥1 kg/m^2 (12.4%) and remained weight stable between -1 to 1 kg/m^2 (11.9%) between pregnancies were LGA compared to 13.5% and 15.9% in women who gained 1-3 and ≥3 kg/m^2 respectively. Overweight women were at lower risk of recurrent LGA in the second pregnancy if they lost ≥1 kg/m^2 (adjusted risk ratio (aRR) 0.69, 95% CI 0.48 to 0.97) whereas overweight women who gained weight (≥3 kg/m^2) were at increased risk of 'new' LGA after having a non-LGA birth in their first pregnancy (aRR 1.35, 95% CI 1.05 to 1.75). Normal-weight women who gained weight were also at increased risk of 'new' LGA in the second pregnancy (aRR 1.26, 95% CI 1.06 to 1.50 with weight gain of 1-3 kg/m^2 and aRR 1.34, 95% CI 1.09 to 1.65 with gain of ≥3 kg/m^2).

Conclusions: Losing weight after an LGA birth reduced the risk of recurrent LGA in the next pregnancy in overweight women, while gaining weight increased LGA risk in women with no previous history of LGA birth. Preventing weight gain between pregnancies is an important prevention measure to achieve better maternal and offspring outcomes.

Article summary

Strengths and limitations of this study

- Utilises antenatal care and birth data from a large population-based cohort including women from all socioeconomic backgrounds
- Objective measurement of both exposure (maternal weight) and outcome in two pregnancies per woman
- Self-reported data for covariables
- Lack of information on breastfeeding and maternal weight gain during pregnancy

For peer review only - http://bmjopen.bmj.com/site/about/guidelines.xhtml

Introduction

The prevalence of maternal obesity has been rising over time. It has more than doubled in England between 1989 and 2007 (7.6% to 15.6%), with the proportion of normal weight pregnancies showing a 12% decrease from 65.6% to 53.6%[1]. Maternal overweight and obesity is a key risk factor for adverse maternal and birth outcomes. It also increases the risk of long-term health problems in the child including obesity, cardiovascular disease, diabetes and cognitive and behavioural disorders[2]. Birthweight is a key early life predictor of long-term health outcomes such as obesity and cardiovascular disease[3] and potentially acts as a mediator on the causal pathway between maternal obesity and long-term offspring outcomes. The incidence of large-for-gestational age (LGA) birth, defined as >90th percentile weight for gestational age, has increased over time in high-income countries[4,5]. LGA is associated with both childhood[6,7] and adult obesity[8-10]. A key risk factor for LGA birth is gestational diabetes (GDM)[11], the incidence of which has also increased over time[12,13]. Offspring of mothers with gestational diabetes have increased risk of childhood overweight and obesity[14,15]. Maternal obesity is an established risk factor for both GDM and LGA birth[16]. Change in maternal body mass index (BMI) between pregnancies could modify the risk of LGA birth in the subsequent pregnancy.

Birthweight, on average, increases with parity. First-born infants tend to have the lowest birthweight among their younger siblings[17-19] up to the fourth pregnancy[20]. However, birthweight was found to decrease with parity for women who had short intervals between their pregnancies (<12 months) while the increase in birthweight with parity was more pronounced in women with long intervals (>24 months)[20]. Also, maternal weight change between pregnancies was found to modify the relationship between parity and birthweight. Women who returned to their pre-pregnancy weight before the next conception had infants who weighed less than infants of women who retained or gained weight between pregnancies[20]. In a UK- based study, women who lost at least six kilograms between their first and second pregnancy had a smaller average increase in birthweight of the second baby compared to women who gained ten kilograms or more (in a 1.60m tall woman, 6 kg equates to approximately 2.3 kg/m^2 and 10 kg to approximately 3.8 kg/m^2)[18].

A large US study showed that women were at an increased risk of having an LGA baby in the second pregnancy if their pre-pregnancy BMI category increased towards overweight or obese between their first and second pregnancies. This applied to all first pregnancy BMI categories, except underweight women who became normal weight by the start of their second pregnancy. Overweight and obese women who dropped BMI category by their second pregnancy remained at an increased risk of LGA birth, but had a lower risk compared to women whose BMI category increased between pregnancies[21].

Another US-based study showed that inter-pregnancy weight gain of ≥2 kg/m^2 in obese women was associated with increased risk of LGA. Weight loss of ≥2 kg/m^2 was associated with a lower adjusted LGA risk compared to the women who maintained their weight within 2 kg/m^2 change between pregnancies[22].

Two studies found a reduced risk of 'new' LGA in the second pregnancy following a non-LGA birth in the first pregnancy with inter-pregnancy weight loss of >1 kg/m^2, and an increased risk with modest (1-3 kg/m^2) and large (≥3 kg/m^2) weight gain. In stratified analysis, the association was stronger in women with a first pregnancy BMI of <25 kg/m^2 [23,24]. A third study only found an increased risk of 'new' LGA in normal weight women who gained ≥4 kg/m^2 between pregnancies and no association in overweight women[25].

To our knowledge, only one study has examined the risk of recurrent LGA (occurring in both first and second pregnancies) in relation to maternal weight change between pregnancies [26]. The study, conducted in Aberdeen, Scotland, included 24520 women of which 813 women had LGA births in both pregnancies. Inter-pregnancy weight gain (≥2 kg/m^2) was associated

For peer review only - http://bmjopen.bmj.com/site/about/guidelines.xhtml

with increased risk of recurrent LGA, while weight loss (≥2 kg/m^2) was protective. Women with BMI <25kg/m^2 were at increased risk of recurrent LGA on gaining weight whereas women with BMI ≥25kg/m^2 were at reduced risk of recurrent LGA on losing weight[26].

In this study, we aimed to investigate the association between the incidence of LGA, recurrent LGA and 'new' LGA births in the second pregnancy and maternal change in BMI between the first and second pregnancies, stratifying by maternal BMI category in the first pregnancy, in a population-based cohort in the South of England.

Methods

This is a population-based cohort of prospectively collected routine healthcare data for antenatal care between January 2003 and September 2017 at University Hospital Southampton, Hampshire, UK. This included all women delivering at this hospital (n= 82098 pregnancies), which is a regional centre for maternity care in and around Southampton. Records of women with their first two consecutive singleton live birth pregnancies were included. Records with unfeasible weight (<30kg), height (>2m) and gestational age (>301 days) values were excluded.

Exposure assessment

Maternal weight in kilograms was routinely measured by a midwife at the first antenatal (booking) appointment of each pregnancy, which is recommended to take place ideally by 10 weeks gestation in the UK, according to the National Institute for Health and Care Excellence Guidelines [27]. Any woman who had a booking appointment at or after 24 weeks of pregnancy was excluded. Height was self-reported. BMI was calculated as weight (in kg) divided by height (in metres) squared.

BMI at the start of the first pregnancy was categorised as underweight (BMI <18.5 kg/m^2), normal weight (18.5 to 24.9 kg/m^2), overweight (25.0 to 29.9 kg/m^2) and obese (≥30 kg/m^2). Change in BMI was calculated as the difference in BMI measured at the booking appointments of the first two consecutive live birth pregnancies for each woman. This change in BMI was then categorised as weight loss (≥1 kg/m^2), weight stable (-1 to 1 kg/m^2) and two categories of weight gain (1- 3 kg/m^2 and ≥3 kg/m^2).

Outcome assessment

Birthweight (grams) was measured by healthcare professionals at birth as part of routine care. Gestational age was based on a dating ultrasound scan which routinely takes place between 10 and 13 weeks gestation[27]. Age- and gender- specific birthweight centiles were calculated using reference values for England and Wales provided in the most recently released national data[28]. Large-for-gestational age was defined as >90th percentile weight for gestational age. This was only defined for babies born between 24 to 42 weeks gestation as reference values only exist for these gestational ages and with determinate gender.

Covariables

Maternal date of birth is recorded at the booking appointment and converted to age (in years) on extraction of the dataset to maintain anonymity. Highest maternal educational

For peer review only - http://bmjopen.bmj.com/site/about/guidelines.xhtml

qualification was self-reported and categorised as primary, secondary, college, undergraduate, postgraduate, graduate and none. For the purposes of this analysis, this was condensed to three categories - secondary (GCSE) and under, college (A levels) and university degree or above. Self-reported ethnicity was recorded under 16 categories and condensed to White, Mixed, Asian, Black/African/Caribbean and Other. Categories of not asked and not stated were coded as missing. Smoking was self-reported as current smoking or non-smoking. Non-smokers were further asked if they had ever smoked or had previously smoked and quit. This was categorised as stopped more than 12 months before conception, stopped less than 12 months before conception or stopped when pregnancy confirmed. Employment status was self-reported at booking appointment and categorised as employed, unemployed, in education, and not specified. Infertility treatment was categorised as no/investigations only and yes (hormonal only, in-vitro fertilisation, gamete intrafallopian transfer and other surgical) in either one or both pregnancies. In this population, an oral glucose tolerance test was used for screening for GDM in women with one or more risk factors (BMI > 30kg/m^2; GDM in previous pregnancy; previous baby weighing $\geq$4.5kg; diabetes in parents or siblings and of Asian, African-Caribbean or Middle Eastern ethnicity)[29]. GDM diagnosis was then reported in the database. Inter-pregnancy interval was defined as the interval between the first live birth and conception of the second pregnancy. The difference in days between two consecutive live births was calculated and gestational age of the latter birth subtracted from this to derive the inter-pregnancy interval.

Statistical analysis

All analysis was performed using Stata 15[30]. Univariable comparisons were carried out using ANOVA for continuous variables and chi square test for categorical variables. Generalised linear regression with log link[31] was used to examine the association between the categorised variable of maternal change in BMI between pregnancies with risk of LGA in the second pregnancy. This was analysed first in the whole sample and then stratified by ‘baseline’ maternal BMI category as calculated in the first antenatal appointment of the first pregnancy.

Risk of LGA in the second pregnancy was explored in the whole sample adjusting for previous pregnancy outcome of LGA. The risk of ‘new’ LGA in second pregnancy after having a non-LGA baby in the first pregnancy was explored in the sub-sample of women who had non-LGA births in the first pregnancy. The risk of recurrent LGA (LGA in both pregnancies) was explored in a sub-sample of women who had LGA births in the first pregnancy.

Initial univariable analysis was followed by multivariable models adjusting for potential confounding factors – maternal age, ethnicity, highest educational qualification, whether or not undergone infertility treatment, employment status, smoking behaviour in second pregnancy, baseline BMI, GDM in second pregnancy and inter-pregnancy interval. Sensitivity analysis was conducted adding gestational age at booking in the second pregnancy to the models.

A statistical significance level of 0.05 with 95% confidence intervals was used in the regression models.

Ethical considerations

All data were fully anonymised by the data holder before being accessed by the research team. Ethics approval was granted by the University of Southampton Faculty of Medicine Ethics Committee: study ID 25508.

For peer review only - http://bmjopen.bmj.com/site/about/guidelines.xhtml

Patient and Public Involvement

Patients and public were not involved in setting the research question or the outcome measures, nor were they involved in developing plans for the design or implementation of the study. However, pregnant woman and mothers of young children have been involved in the planning stages of a research project building on this analysis.

Results

The first and second pregnancies of 15940 women were included. Of these, 16.0% of women lost ≥1 kg/m², 36.3% remained weight stable (-1 to 1 kg/m²), 27.9% gained 1-3 kg/m² and 19.8% gained ≥3 kg/m² between their first and second live birth pregnancies. Weight loss of >2 kg/m² was observed in 7.3% of women whereas 10.7% gained >2 kg/m². Mean BMI at second pregnancy booking was 30.8 kg/m² (standard deviation (SD) 5.9) in women who gained ≥3 kg/m², 25.9 kg/m² (SD 4.7) in women who gained 1-3kg/m² , 24.1 kg/m² (SD 5.1) in women who lost weight, and 23.8 kg/m² (SD 4.4) women whose weight remained stable between pregnancies ($p<0.001$) (Table 1).

Women who gained ≥3 kg/m² by the start of their second pregnancy were more likely to be smokers, unemployed, with lower educational attainment and to have a longer inter-pregnancy interval, compared to those who maintained a stable weight between pregnancies. Mean maternal age was lowest in the women who gained ≥3 kg/m² (27.3 years, SD 5.5) and highest in the women who remained weight stable (29.8 years, SD 5.3). Mean maternal age in women who lost weight was 28.7 years (SD 5.4).

Mothers who gained ≥3 kg/m² were more likely to be obese (48.3%) at the start of the second pregnancy compared to 16.1% in women who gained 1-3 kg/m², 9.2% in women who remained weight stable and 11.9% in women who lost ≤1 kg/m².

A lower proportion of babies born to women who lost weight (12.4%) or remained weight stable (11.9%) between pregnancies were LGA compared to 13.5% in women who gained 1-3 kg/m² and 15.9% in women who gained ≥3 kg/m² ($p<0.001$) (Table 1, Figure 1). Compared to normal weight women, overweight and obese women were at increased risk of LGA births in both pregnancies with risk highest in obese women (unadjusted relative risk (RR) 2.06, 95% CI 1.78 to 2.38 and 1.86, 95% CI 1.69 to 2.05 in first and second pregnancy respectively). Figure 2 shows the percentage of all LGA as recurrent LGA or 'new' LGA in second pregnancy by the inter-pregnancy change in maternal BMI stratified by maternal BMI category calculated at the start of the first pregnancy. The lowest proportion of LGA births in the second pregnancy was in underweight women in the first pregnancy who remained weight stable (2.8%), while the highest was in obese women who gained ≥3 kg/m² (21.2%). Within BMI categories, recurrent LGA was lowest in normal weight and overweight women who lost weight and highest in obese women who gained 1-3 kg/m².

Women who gained ≥3 kg/m² were at increased risk of LGA in the second pregnancy in the full sample compared to remaining weight stable (aRR 1.28, 95% CI 1.14 to 1.44) (Figure 1). There was a significantly reduced risk of recurrent LGA birth in the second pregnancy in overweight women who had a LGA infant in the first pregnancy and lost ≥1 kg/m² in weight (aRR 0.69, 95% CI 0.48 to 0.97) (Table 2). No association was observed between risk of recurrent LGA and maternal BMI change between pregnancies in underweight, normal weight and obese women.

For peer review only - http://bmjopen.bmj.com/site/about/guidelines.xhtml

There was an increased risk of 'new' LGA birth in the second pregnancy after having a non-LGA infant in the first pregnancy in normal weight women who gained 1-3 kg/m^2 (aRR 1.26, 95% CI 1.06 to 1.50) and in normal weight and overweight women who had gained ≥3 kg/m^2 weight (aRR 1.34, 95% CI 1.09 to 1.65, aRR 1.35, 95% CI 1.05 to 1.75, respectively) (Table 3). No association was observed between the risk of 'new' LGA in the second pregnancy and maternal BMI inter-pregnancy change in obese women.

Discussion

This study examined the association between change in women's BMI between their first and second live birth pregnancies and risk of LGA birth in the second pregnancy in a population-based cohort of 15940 women in the South of England. Almost half of the sample (48%) of women gained ≥1 kg/m^2 in the time between the first antenatal care visits during their first and second pregnancies. The proportion of LGA births was significantly higher in women with an inter-pregnancy weight gain of ≥3 kg/m^2 (16%) compared to women who lost weight (12%) and those who remained weight stable (12%) between pregnancies. Overweight women who lost ≥1 kg/m^2 had a reduced risk of recurrent LGA. Normal weight women who gained 1–3 kg/m^2 and both normal weight and overweight women who gained ≥3 kg/m^2 between pregnancies had an increased risk of LGA birth in their second pregnancy after a non-LGA birth in the first.

Compared to the population-based Swedish cohort which carried out a similar analysis for LGA and other outcomes in 151025 women using data from 1992 to 2001, a lower proportion of women remained weight stable in our cohort (46% compared to 36%) and a higher proportion lost (11% compared to 16%) or gained (43% compared to 48%) weight. Amongst women who gained weight, a higher proportion gained ≥3 kg/m^2 in this cohort (20%) compared to the Swedish cohort (11%)[23]. Similarly, in comparison to a population-based cohort of 24520 women in Aberdeen, Scotland; for the period 1986 to 2013, a larger proportion of women in our study both lost and gained weight[26]. The differences could reflect the increase in the prevalence of maternal overweight and obesity over time since our data are more recent.

In the adjusted model utilising the full sample, we showed an increased risk of LGA in the second pregnancy for inter-pregnancy weight gain compared to weight remaining stable. In a population-based cohort in the US, women were found to be at increased risk of LGA in the second pregnancy if their pre-pregnancy BMI category changed towards overweight or obese from first to second pregnancy regardless of their BMI category in first pregnancy except in underweight women who increased to normal weight[21]. This study is different to ours in that it only examined risk in second pregnancy without adjustment for LGA outcome in first pregnancy. It also considered weight change as change in BMI category only, while we studied change in maternal BMI regardless of whether BMI category has changed or not in the second pregnancy.

In obese women in the US, inter-pregnancy weight gain of ≥2 kg/m^2 was associated with increased risk of LGA and a weight loss of ≥2 kg/m^2 was associated with decreased risk compared to the reference group of weight maintained (between >-2 and <2 kg/m^2)[22]. We found no association between weight change and risk of second pregnancy LGA in women who were obese at the start of their first pregnancy. This may be because obese women are already at increased risk of LGA births, and the average inter-pregnancy BMI change in this subgroup was not large

For peer review only - http://bmjopen.bmj.com/site/about/guidelines.xhtml

enough to detect a further increase in risk. Greater efforts are needed for primary prevention of obesity in women of child bearing age and obese women need more effective weight loss strategies in inter-partum period to assess impact on LGA and other outcomes.

Risk of recurrent LGA was analysed in one previous study in Scotland which found that inter-pregnancy weight gain (≥2 kg/m^2) was associated with increased risk of recurrent LGA. In that study, weight loss (≥2 kg/m^2) was associated with reduced LGA risk. Stratification by first pregnancy BMI showed that women with BMI <25kg/m^2) were at increased risk of recurrent LGA on gaining ≥2 kg/m^2 whereas women with BMI ≥25kg/m^2 were at reduced risk of recurrent LGA on losing ≥2 kg/m^2 weight[26]. We showed a similar reduction in risk in overweight women who lost ≥1 BMI unit between pregnancies, but found no association in normal weight women. This difference in findings may be because the <25kg/m^2 group in the previous Scottish study included underweight women whereas our stratified analysis examined normal weight women separately to underweight women.

We showed an increased risk of 'new' LGA in the second pregnancy (after a non-LGA birth in the first pregnancy) with inter-pregnancy weight gain compared to remaining weight stable. After stratification by BMI, we found that this association between inter-pregnancy weight gain and new LGA remained only in normal-weight and overweight women. The findings from this study are in line with findings with other studies in Scotland[24] and Sweden[23] which found increased risk of 'new' LGA with modest (1-3 kg/m^2) and large (≥3 kg/m^2) weight gain. Both studies also found a decreased risk with inter-pregnancy weight loss of >1 kg/m^2 which was not found in our study. Both studies stratified BMI as < and ≥25kg/m^2, while we further stratified the ≥25kg/m^2 category as overweight (BMI 25-29.9kg/m^2) and obese (≥30kg/m^2) and found an increased risk of 'new' LGA in overweight, but not in obese women. We carried out sensitivity analysis merging overweight and obese categories and found increased risk in this category (data not shown) suggesting that the results are comparable to previous studies.

Women included in this analysis had a range of inter-pregnancy interval of less than 1 to up to 12 years and thus weight change could be due to postpartum weight retention or late postpartum weight gain. There is evidence that women who do not lose pregnancy weight at one year postpartum are more likely to retain weight longer term[32]. We examined the risk of maternal inter-pregnancy weight gain with length of the inter-pregnancy interval and found that women with an interval of 12-23 months were least likely to start the next pregnancy at a higher weight[33]. We also examined the length of the inter-pregnancy interval as a predictor for LGA risk adjusting for inter-pregnancy weight change and found no association[34].

The Development Origins of Health and Disease concept suggests that adverse exposures during development could lead to enhanced susceptibility in the foetus thus increasing the risk of non-communicable diseases in later life. Although the focus has previously been on exposures during pregnancy, the importance of the preconception period is now recognised[35-37]. Efforts to systematically identify women in the preconception period to improve health and lifestyle during conception are underway[37]. Promoting health of all women of child-bearing age with targeting of women and partners planning a pregnancy has been identified as an effective approach to improving preconception health[36]. It is difficult to identify all women who are planning a pregnancy but as the inter-conception period is also the preconception period for the next pregnancy, it is important to engage with women during this period to optimise their and their children's health.

Future research that characterises the predictors of postpartum weight change would help design interventions to support postpartum weight loss and prevent weight gain. Key to this is an understanding of the pattern of weight change during this period as well as identifying the optimal setting and delivery of the intervention. Support with healthy eating and physical activity is more commonly received during pregnancy than after birth. Even when lifestyle

For peer review only - http://bmjopen.bmj.com/site/about/guidelines.xhtml

advice is received postpartum, it was found not to be associated with healthy diet or physical activity behaviours[38]. Most interventions that have been successful in limiting and promoting postpartum weight loss were combined diet and physical activity interventions with self-monitoring[39]. However, the timing of engaging women and length of intervention or engagement are important with one study showing that an intervention from 16 weeks pregnancy to six months postpartum was more effective than the same intervention from birth to six months postpartum intervention[40].

As pregnancy and early postpartum is a period of major change for women and their families, interventions need to be carefully designed to be attractive, flexible, affordable and feasible for women at this stage with competing priorities and time demands. Focus during the postpartum period in the UK healthcare system is mostly on child health and development. The feasibility and effectiveness of better utilising contact time with health professionals during the two years after birth to engage and support maternal health needs to be explored. There may also be a role for mutual support groups for mothers. There is additionally a need to recognise that weight management issues are greater in more disadvantaged mothers so there is also the issue of identifying the most effective weight management strategies for such mothers to reduce social inequity in subsequent birth and maternal outcomes. Weight gain does not occur in isolation and usually combined with other risk factors particularly in socioeconomically disadvantaged groups and hence a holistic approach taking into account priority setting for these families should be considered.

Strengths and limitations

This is a relatively large population-based cohort including women from all socioeconomic and ethnic backgrounds delivering at a large maternity centre in Southampton, UK, thus representative of the regional population. According to the UK Department of Communities and Local Government English indices of deprivation report, Southampton is more deprived than average with the situation having worsened between 2010 and 2015[41]. However, about half of the women included in this analysis reside in the rest of Hampshire (the region where Southampton is situated), which is less deprived. Our sample was 87% of White ethnicity, which is comparable to the 2011 England and Wales population census of 86% White[42]. The analysis was adjusted for several key confounders that were reasonably complete (96% complete for ethnicity and employment status). Both the maternal weight (used to calculate exposure) and birthweight in this study were objectively measured by healthcare professionals as part of routine antenatal and delivery care.

An important limitation was the lack of information on gestational weight gain during pregnancy, breastfeeding and paternal characteristics/behaviour, which are potential confounders in the association between maternal inter-pregnancy weight gain and LGA birth[43]. Women who had their first booking appointment later into the pregnancy (more than 24 weeks) were excluded from the analysis in order to ensure comparability of weight measurements between pregnancies. We also adjusted for gestational age at booking, as this was the point when maternal BMI was measured, in sensitivity analysis and the estimates remained similar. Some of the confounding factors which were accounted for in the analysis were self-reported, however the information was collected prospectively, therefore any measurement error in likely to be non-differential. Another limitation is that these findings are based on observational data so inferences about causation cannot be drawn and the risk of residual confounding influencing the results needs to be considered.

In conclusion, maternal weight gain of 1 or more kg/m^2 between first and second pregnancy had a prevalence of 48%, and it was associated with risk of LGA in the second pregnancy in this English cohort. Risk of 'new' LGA was higher in normal weight and overweight women who gained weight after a non-LGA birth in their first pregnancy compared to those who remained weight stable. Overweight women were at a lower risk of a recurrent LGA birth in

For peer review only - http://bmjopen.bmj.com/site/about/guidelines.xhtml

their second pregnancy if they lost weight between pregnancies. Greater efforts are needed for primary prevention of overweight and obesity in women of child bearing age. Supporting efforts to lose weight in overweight and obese women between pregnancies, and stop weight gain in all women planning to have further children (except those who are underweight) are important preventive measures of subsequent adverse maternal and offspring health outcomes.

Author Contributions

Study design (NZ, PJR, NSM, NAA), data analysis (NZ, SW), acquisition and interpretation of the data (NZ, NAA), drafting of the abstract (NZ), revising for content (NZ, SW, PJR, NSM, NAA) and approval of final version before submission (NZ, SW, PJR, NSM, NAA).

Data statement

Anonymised data are only available upon request from the authors conditional on approval of the appropriate institutional ethics and research governance processes.

Acknowledgements

We would like to thank David Cable (Electronic Patient Records Implementation and Service Manager) and Florina Borca (Senior Information Analyst R&D) at University Hospital Southampton for support in accessing the data used in this study. We also thank clinical colleagues at the Princess Anne Hospital, Southampton for collecting the data.

Funding

This work is supported by a University of Southampton Primary Care and Population Sciences PhD studentship (to NZ) , the Academy of Medical Sciences and the Wellcome Trust (Grant no: AMS_HOP001\1060 to NAA). NAA is also in receipt of research support from the National Institute for Health Research through the NIHR Southampton Biomedical Research Centre.

Competing interests

The authors have no competing interests to declare.

References

1. Heslehurst N, Rankin J, Wilkinson JR, et al. A nationally representative study of maternal obesity in England, UK: trends in incidence and demographic inequalities in 619 323 births, 1989-2007. *Int J Obes (Lond)* 2010;34(3):420-8.
2. Poston L. Maternal obesity, gestational weight gain and diet as determinants of offspring long term health. *Best Pract Res Clin Endocrinol Metab* 2012;26(5):627-39.
3. Barker DJP. Fetal origins of coronary heart disease. *BMJ* 1995;311:171-74.

For peer review only - http://bmjopen.bmj.com/site/about/guidelines.xhtml

4. Kramer MS, Morin I, Yang H, et al. Why are babies getting bigger? Temporal trends in fetal growth and its determinants. *J Pediatr* 2002;141(4):538-42.
5. Surkan PJ, Hsieh CC, Johansson AL, et al. Reasons for increasing trends in large for gestational age births. *Obstet Gynecol* 2004;104(4):720-6.
6. Reilly JJ, Armstrong J, Dorosty AR, et al. Early life risk factors for obesity in childhood: cohort study. *BMJ* 2005;330(7504):1357.
7. Danielzik S, Czerwinski-Mast M, Langnase K, et al. Parental overweight, socioeconomic status and high birth weight are the major determinants of overweight and obesity in 5-7 y-old children: baseline data of the Kiel Obesity Prevention Study (KOPS). *Int J Obes Relat Metab Disord* 2004;28(11):1494-502.
8. Curhan GC, Willett WC, Rimm EB, et al. Birth Weight and Adult Hypertension, Diabetes Mellitus, and Obesity in US Men. *Circulation* 1996;94:3246-50.
9. Curhan GC, Chertow GM, Willett WC, et al. Birth Weight and Adult Hypertension and Obesity in Women. *Circulation* 1996;94:1310-15.
10. Phillips DIW, Young JB. Birth weight, climate at birth and the risk of obesity in adult life. *International Journal of Obesity* 2000;24:281-87.
11. Casey BM, Lucas MJ, McIntire DD, et al. Pregnancy Outcomes in Women With Gestational Diabetes Compared With the General Obstetric Population. *Obstetrics & Gynecology* 1997;90(6):869-73.
12. Hunt KJ, Schuller KL. The Increasing Prevalence of Diabetes in Pregnancy. *Obstet Gynecol Clin North Am.* 2007;34(2):173-99.
13. Ignell C, Claesson R, Anderberg E, et al. Trends in the prevalence of gestational diabetes mellitus in southern Sweden, 2003-2012. *Acta Obstet Gynecol Scand* 2014;93(4):420-4.
14. Baptiste-Roberts K, Nicholson WK, Wang NY, et al. Gestational diabetes and subsequent growth patterns of offspring: the National Collaborative Perinatal Project. *Matern Child Health J* 2012;16(1):125-32.
15. Zhu Y, Olsen SF, Mendola P, et al. Growth and obesity through the first 7 y of life in association with levels of maternal glycemia during pregnancy: a prospective cohort study. *Am J Clin Nutr* 2016;103(3):794-800.
16. Marchi J, Berg M, Dencker A, et al. Risks associated with obesity in pregnancy, for the mother and baby: a systematic review of reviews. *Obes Rev* 2015;16(8):621-38.
17. Swamy GK, Edwards S, Gelfand A, et al. Maternal age, birth order, and race: differential effects on birthweight. *J Epidemiol Community Health* 2012;66(2):136-42.
18. Wilcox MA, Chang AMZ, Johnson IR. The effects of parity on birthweight using successive pregnancies. *Acta Obstetricia et Gynecologica Scandinavica* 1996;75(5):459-63.
19. Beaty TH, Skjaerven R, Breazeale DR, et al. Analyzing Sibship Correlations in Birth Weight Using Large Sibships From Norway. *Genetic Epidemiology* 1997;14:423-33.
20. Hinkle SN, Albert PS, Mendola P, et al. The association between parity and birthweight in a longitudinal consecutive pregnancy cohort. *Paediatr Perinat Epidemiol* 2014;28(2):106-15.
21. Getahun D, Ananth CV, Peltier MR, et al. Changes in prepregnancy body mass index between the first and second pregnancies and risk of large-for-gestational-age birth. *Am J Obstet Gynecol* 2007;196(6):530 e1-8.
22. Jain AP, Gavard JA, Rice JJ, et al. The impact of interpregnancy weight change on birthweight in obese women. *Am J Obstet Gynecol* 2013;208(3):205 e1-7.
23. Villamor E, Cnattingius S. Interpregnancy weight change and risk of adverse pregnancy outcomes: a population-based study. *Lancet* 2006;368(9542):1164-70.
24. Wallace JL, Bhattacharya S, Campbell DM, et al. Inter-pregnancy weight change impacts placental weight and is associated with the risk of adverse pregnancy outcomes in the second pregnancy. *BMC Pregnancy and Childbirth* 2014;14(40).
25. McBain RD, Dekker GA, Clifton VL, et al. Impact of inter-pregnancy BMI change on perinatal outcomes: a retrospective cohort study. *Eur J Obstet Gynecol Reprod Biol* 2016;205:98-104.

For peer review only - http://bmjopen.bmj.com/site/about/guidelines.xhtml

26. Wallace JM, Bhattacharya S, Campbell DM, et al. Inter-Pregnancy Weight Change and the Risk of Recurrent Pregnancy Complications. *PLoS One* 2016;11(5):e0154812.
27. National Institute for Health Care and Excellence. Antenatal care for uncomplicated pregnancies. Manchester, UK, 2008.
28. Norris T, Seaton SE, Manktelow BN, et al. Updated birth weight centiles for England and Wales. *Archives of Disease in Childhood. Fetal and Neonatal Edition* 2017.
29. National Health Service. *Gestational Diabetes*. https://www.nhs.uk/conditions/gestational-diabetes/ (accessed 26/11/2018).
30. Stata Statistical Software: Release 15 [program]. College Station, TX: StataCorp LLC, 2017.
31. Cummings P. Methods for estimating adjusted risk ratios. *The Stata Journal* 2009;9(2):175-96.
32. Linné Y, Dye L, Barkeling B, et al. Long-Term Weight Development in Women: A 15-Year Follow-up of the Effects of Pregnancy. *Obesity Research* 2004;12(7):1166-78.
33. Ziauddeen N, Roderick PJ, Macklon NS, et al. Length of inter-pregnancy interval and subsequent preconception adiposity: Findings from a population-based cohort in the South of England. *Revue d'Épidémiologie et de Santé Publique* 2018;66:S374.
34. Ziauddeen N, Roderick PJ, Macklon NS, et al. LB5 Is the duration of the preceding inter-pregnancy interval associated with offspring's size at birth? – analysis of a UK population-based cohort. *Journal of Epidemiology and Community Health* 2018;72(Suppl 1):A43.
35. Fleming TP, Watkins AJ, Velazquez MA, et al. Origins of lifetime health around the time of conception: causes and consequences. *The Lancet* 2018;391(10132):P1842-52.
36. Barker M, Dombrowski SU, Colbourn T, et al. Intervention strategies to improve nutrition and health behaviours before conception. *The Lancet* 2018;391(10132):P1853-64.
37. Stephenson J, Heslehurst N, Hall H, et al. Before the beginning: nutrition and lifestyle in the preconception period and its importance for future health. *The Lancet* 2018;391(10132):P1830-41.
38. van der Pligt P, Olander EK, Ball K, et al. Maternal dietary intake and physical activity habits during the postpartum period: associations with clinician advice in a sample of Australian first time mothers. *BMC Pregnancy Childbirth* 2016;16:27.
39. van der Pligt P, Willcox J, Hesketh KD, et al. Systematic review of lifestyle interventions to limit postpartum weight retention: implications for future opportunities to prevent maternal overweight and obesity following childbirth. *Obes Rev* 2013;14(10):792-805.
40. Huang TT, Yeh CY, Tsai YC. A diet and physical activity intervention for preventing weight retention among Taiwanese childbearing women: a randomised controlled trial. *Midwifery* 2011;27(2):257-64.
41. Department for Communities and Local Government. The English Indices of Deprivation 2015, 2015.
42. Office for National Statistics. Ethnicity and National Identity in England and Wales 201, 2012.
43. Nohr EA, Vaeth M, Baker JL, et al. Combined associations of prepregnancy body mass index and gestational weight gain with the outcome of pregnancy. *American Journal of Clinical Nutrition* 2008;87(6):1750-59.

Figure legends

Figure 1: The percentage and risk of LGA births in second pregnancy stratified by maternal inter-pregnancy weight change categories

Figure 2: The absolute percentage of LGA births in second pregnancy by inter-pregnancy change in maternal body mass (BMI) stratified by maternal BMI category in the first pregnancy and previous outcome of LGA

For peer review only - http://bmjopen.bmj.com/site/about/guidelines.xhtml

Table legends:

Table 1: Maternal and birth characteristics in the second live birth pregnancy categorised by maternal weight change from the first livebirth pregnancy for the period of January 2003 - September 2017, University Hospital Southampton NHS Foundation Trust, Hampshire, England

Table 2: Associations between risk of recurrent large-for-gestational age (LGA) birth in the second pregnancy and change in maternal body mass index (BMI) between pregnancies as measured at the first antenatal visit of each pregnancy stratified by BMI category in the first pregnancy

Table 3: Associations between the risk of ‘new’ large-for-gestational age (LGA) birth in the second pregnancy following a non-LGA birth in the first pregnancy and change in maternal body mass index (BMI) between pregnancies measured at the first antenatal visit of each pregnancy stratified by BMI category in the first pregnancy

For peer review only

Table 1: Maternal and birth characteristics in the second live birth pregnancy categorised by maternal weight change gain from the first live birth pregnancy for the period of January 2003 - September 2017, University Hospital Southampton NHS Foundation Trust, Hampshire, England

	Lost ≤ -1 kg/m² from previous pregnancy	Weight stable (>-1 to <1 kg/m²)	Gained 1-3 kg/m² from previous pregnancy	Gained ≥3 kg/m² from previous pregnancy	p*
N	2548	5785	4446	3161	
Maternal age, years (mean ± SD)	28.7 ± 5.4	29.8 ± 5.3	29.2 ± 5.4	27.3 ± 5.5	<0.001
Timing of first booking appointment, weeks (mean ± SD)	10.8 ± 2.3	11.0 ± 2.3	11.1 ± 2.4	11.0 ± 2.6	<0.001
Maternal BMI at booking, kg/m² (mean ± SD)	24.1 ± 5.1	23.8 ± 4.4	25.9 ± 4.7	30.8 ± 5.9	<0.001
Maternal BMI at booking in first pregnancy (%, 95% CI)					
Underweight (< 18.5)	0.8 (0.5 to 1.2)	4.3 (3.8 to 4.8)	5.3 (4.7 to 6.0)	3.7 (3.1 to 4.4)	<0.001
Normal weight (18.5 to 24.9)	47.6 (45.6 to 49.5)	67.4 (66.2 to 68.6)	62.5 (61.0 to 63.9)	49.0 (47.2 to 50.7)	
Overweight (25.0 to 29.9)	30.1 (28.3 to 31.9)	19.4 (18.4 to 20.5)	22.0 (20.8 to 23.3)	29.5 (28.0 to 31.2)	
Obese (≥30.0)	21.5 (19.9 to 23.2)	8.9 (8.2 to 9.7)	10.2 (9.3 to 11.1)	17.8 (16.5 to 19.2)	
Maternal BMI at booking in second pregnancy (%, 95% CI)					
Underweight (< 18.5)	6.9 (5.9 to 7.9)	4.3 (3.8 to 4.8)	0.6 (0.4 to 0.9)	0.0 (0.0 to 0.2)	<0.001
Normal weight (18.5 to 24.9)	61.1 (59.2 to 63.0)	66.8 (65.6 to 68.1)	50.7 (49.2 to 52.1)	14.9 (13.7 to 16.2)	
Overweight (25.0 to 29.9)	20.1 (18.6 to 21.7)	19.7 (18.7 to 20.7)	32.6 (31.2 to 34.0)	36.7 (35.0 to 38.4)	
Obese (≥30.0)	11.9 (10.7 to 13.3)	9.2 (8.5 to 10.0)	16.1 (15.0 to 17.2)	48.3 (46.6 to 50.1)	
Maternal smoking status at booking (%, 95% CI)					
Never smoked/quit	57.2 (55.3 to 59.2)	63.0 (61.8 to 64.3)	60.5 (59.0 to 62.0)	50.7 (48.9 to 52.4)	<0.001
Stopped >1 year before conceiving	16.1 (14.6 to 17.5)	17.2 (16.3 to 18.2)	17.7 (16.5 to 18.8)	14.9 (13.7 to 16.2)	
Stopped <1 year prior to conceiving	4.0 (3.3 to 4.8)	2.8 (2.4 to 3.2)	3.5 (3.0 to 4.1)	4.9 (4.2 to 5.7)	
Stopped when pregnancy confirmed	6.8 (5.8 to 7.8)	5.9 (5.3 to 6.6)	6.9 (6.2 to 7.7)	10.3 (9.3 to 11.4)	
Continued smoking	15.9 (14.5 to 17.4)	11.0 (10.2 to 11.8)	11.4 (10.5 to 12.4)	19.1 (17.8 to 20.6)	
Maternal education (%, 95% CI)					
Secondary (GCSE) or under	30.7 (28.9 to 32.5)	24.0 (22.9 to 25.2)	29.4 (28.1 to 30.8)	36.3 (34.6 to 38.0)	<0.001
College (A levels)	40.4 (38.5 to 42.3)	38.8 (37.6 to 40.1)	39.5 (38.1 to 41.0)	45.8 (44.0 to 47.5)	

For peer review only - http://bmjopen.bmj.com/site/about/guidelines.xhtml

University degree or above	28.9 (27.2 to 30.7)	37.1 (35.9 to 38.4)	31.1 (29.7 to 32.5)	17.9 (16.6 to 19.3)	
Maternal employment (%, 95% CI)					
Employed	66.2 (64.3 to 68.0)	71.7 (70.5 to 72.9)	67.2 (65.8 to 68.5)	56.5 (54.8 to 58.2)	<0.001
Unemployed	31.8 (30.0 to 33.7)	26.9 (25.8 to 28.1)	31.1 (29.7 to 32.5)	41.6 (39.8 to 43.3)	
In education	0.9 (0.6 to 1.4)	0.8 (0.6 to 1.1)	1.1 (0.8 to 1.4)	1.3 (0.9 to 1.8)	
Not specified	1.0 (0.7 to 1.5)	0.6 (0.4 to 0.8)	0.7 (0.5 to 1.0)	0.6 (0.4 to 1.0)	
Ethnicity (%, 95% CI)					
White	89.9 (88.7 to 91.1)	88.0 (87.1 to 88.8)	85.1 (84.0 to 86.1)	84.8 (83.5 to 86.1)	<0.001
Mixed	0.8 (0.5 to 1.3)	0.9 (0.7 to 1.2)	1.4 (1.1 to 1.8)	1.6 (1.1 to 2.0)	
Asian	4.8 (4.0 to 5.7)	5.6 (5.0 to 6.0)	7.2 (6.5 to 8.0)	7.7 (6.8 to 8.7)	
Black/African/Caribbean	0.6 (0.4 to 1.0)	1.0 (0.8 to 1.3)	1.6 (1.3 to 2.1)	2.4 (1.9 to 3.0)	
Other	0.7 (0.4 to 1.1)	1.0 (0.8 to 1.3)	1.0 (0.8 to 1.4)	1.3 (0.9 to 1.7)	
Not specified	3.1 (2.5 to 3.9)	3.5 (3.0 to 4.0)	3.6 (3.1 to 4.2)	2.2 (1.8 to 2.8)	
Inter-pregnancy interval (median, IQR)	21.7 (14.4 to 32.7)	21.6 (14.1 to 32.0)	23.7 (14.4 to 35.6)	27.7 (16.0 to 45.6)	<0.001
Inter-pregnancy interval (%, 95% CI)					
0-11 months	17.4 (15.9 to 18.9)	17.6 (16.6 to 18.6)	18.1 (17.0 to 19.3)	16.6 (15.4 to 17.9)	<0.001
12-23 months	39.8 (37.8 to 41.7)	39.9 (38.6 to 41.1)	33.1 (31.7 to 34.5)	26.3 (24.8 to 27.9)	
24-35 months	22.6 (21.0 to 24.2)	23.6 (22.5 to 24.7)	24.4 (23.2 to 25.7)	20.5 (19.1 to 21.9)	
36 months or more	20.3 (18.7 to 21.9)	18.9 (17.9 to 20.0)	24.3 (23.1 to 25.6)	36.5 (34.9 to 38.2)	
Birthweight, grams (mean ± SD)	3463 ± 563	3467 ± 523	3507 ± 536	3531 ± 558	
Previous size at birth (first pregnancy)					
Small-for-gestational age	13.1 (11.8 to 14.4)	12.6 (11.8 to 13.5)	11.7 (10.8 to 12.7)	12.4 (11.3 to 13.6)	0.11
Appropriate-for-gestational age	79.6 (77.9 to 81.1)	81.1 (80.0 to 82.1)	81.2 (80.1 to 82.4)	79.9 (78.4 to 81.3)	
Large-for-gestational age	7.4 (6.4 to 8.5)	6.3 (5.7 to 7.0)	7.1 (6.3 to 7.8)	7.7 (6.8 to 8.7)	
Size at birth (second pregnancy)					
Small-for-gestational age	8.7 (7.6 to 9.8)	7.0 (6.4 to 7.7)	6.2 (5.5 to 6.9)	6.7 (5.9 to 7.6)	<0.001
Appropriate-for-gestational age	79.0 (77.3 to 80.5)	81.1 (80.0 to 82.1)	80.3 (79.1 to 81.5)	77.4 (75.9 to 78.9)	
Large-for-gestational age	12.4 (11.1 to 13.7)	11.9 (11.1 to 12.8)	13.5 (12.5 to 14.5)	15.9 (14.6 to 17.2)	

*p values calculated using ANOVA for continuous and chi square test for categorical variables

For peer review only - http://bmjopen.bmj.com/site/about/guidelines.xhtml

Table 2: Associations between risk of recurrent large-for-gestational age (LGA) birth in the second pregnancy and change in maternal body mass index (BMI) between pregnancies as measured at the first antenatal visit of each pregnancy stratified by BMI category in the first pregnancy

Maternal BMI change (categorised)		Full sample			Normal weight at first pregnancy			Overweight at first pregnancy			Obese at first pregnancy		
		n	Relative risk, (RR)*	95% CI	n	RR*	95% CI	n	RR*	95% CI	n	RR*	95% CI
Lost ≤ -1 kg/m^2 from previous pregnancy	Unadjusted	1109	0.89	0.74 to 1.08	521	0.80	0.54 to 1.20	338	**0.68**	**0.50 to 0.94**	236	1.16	0.79 to 1.69
	Adjusted**	1066	0.88	0.72 to 1.07	500	0.79	0.54 to 1.17	324	**0.69**	**0.48 to 0.97**	229	1.21	0.79 to 1.83
Weight stable (>-1 to <1 kg/m^2)			Ref			Ref			Ref			Ref	
Gained 1-3 kg/m^2 from previous pregnancy	Unadjusted		0.97	0.83 to 1.13		0.97	0.78 to 1.21		0.81	0.62 to 1.06		1.25	0.85 to 1.83
	Adjusted**		0.98	0.84 to 1.15		1.02	0.83 to 1.27		0.81	0.61 to 1.08		1.28	0.86 to 1.91
Gained ≥3 kg/m^2 from previous pregnancy	Unadjusted		0.96	0.81 to 1.14		0.89	0.68 to 1.17		0.87	0.66 to 1.14		1.16	0.79 to 1.71
	Adjusted**		1.00	0.83 to 1.20		0.91	0.68 to 1.21		0.91	0.67 to 1.25		1.28	0.84 to 1.94

*Generalised linear model with log link and robust variance estimator used to derive RR

**Adjusted for: maternal age, ethnicity, highest educational qualification, whether undergone infertility treatment, smoking status, employment status, baseline BMI, gestational diabetes in current pregnancy and inter-pregnancy interval

For peer review only - http://bmjopen.bmj.com/site/about/guidelines.xhtml

Table 3: Associations between the risk of 'new' large-for-gestational age (LGA) birth in the second pregnancy following a non-LGA birth in the first pregnancy and change in maternal body mass index (BMI) between pregnancies measured at the first antenatal visit of each pregnancy stratified by BMI category in the first pregnancy

Maternal BMI change (categorised)		Full sample			Underweight at first pregnancy			Normal weight at first pregnancy			Overweight at first pregnancy			Obese at first pregnancy		
		n	Relative risk, (RR)*	95% CI	n	RR*	95% CI	n	RR*	95% CI	n	RR*	95% CI	n	RR*	95% CI
Lost ≤ -1 kg/m² from previous pregnancy	Unadjusted	14788	1.05	0.91 to 1.22	606	-	-	8888	0.88	0.68 to 1.14	3458	0.95	0.73 to 1.24	1836	0.90	0.67 to 1.23
	Adjusted**	14215	0.94	0.80 to 1.10		-	-	8513	0.87	0.68 to 1.12	3326	0.96	0.72 to 1.29		0.95	0.67 to 1.34
Weight stable (>-1 to <1 kg/m²)			Ref			Ref			Ref			Ref			Ref	
Gained 1-3 kg/m² from previous pregnancy	Unadjusted		**1.16**	**1.03 to 1.31**		1.21	0.45 to 3.29		**1.20**	**1.02 to 1.40**		1.19	0.94 to 1.50		0.89	0.65 to 1.23
	Adjusted**		1.13	0.99 to 1.28		1.04	0.36 to 3.04		**1.26**	**1.06 to 1.50**		1.16	0.89 to 1.50		0.86	0.61 to 1.22
Gained ≥3 kg/m² from previous pregnancy	Unadjusted		**1.40**	**1.24 to 1.59**		**2.83**	**1.08 to 7.40**		**1.37**	**1.14 to 1.64**		1.22	0.96 to 1.53		1.10	0.82 to 1.46
	Adjusted**		**1.34**	**1.17 to 1.54**		2.08	0.67 to 6.51		**1.34**	**1.09 to 1.65**		**1.35**	**1.05 to 1.75**		1.21	0.89 to 1.65

*Generalised linear model with log link and robust variance estimator used to derive RR

**Adjusted for: maternal age, ethnicity, highest educational qualification, whether undergone infertility treatment, smoking status, employment status, baseline BMI, gestational diabetes in current pregnancy and inter-pregnancy interval

For peer review only - http://bmjopen.bmj.com/site/about/guidelines.xhtml

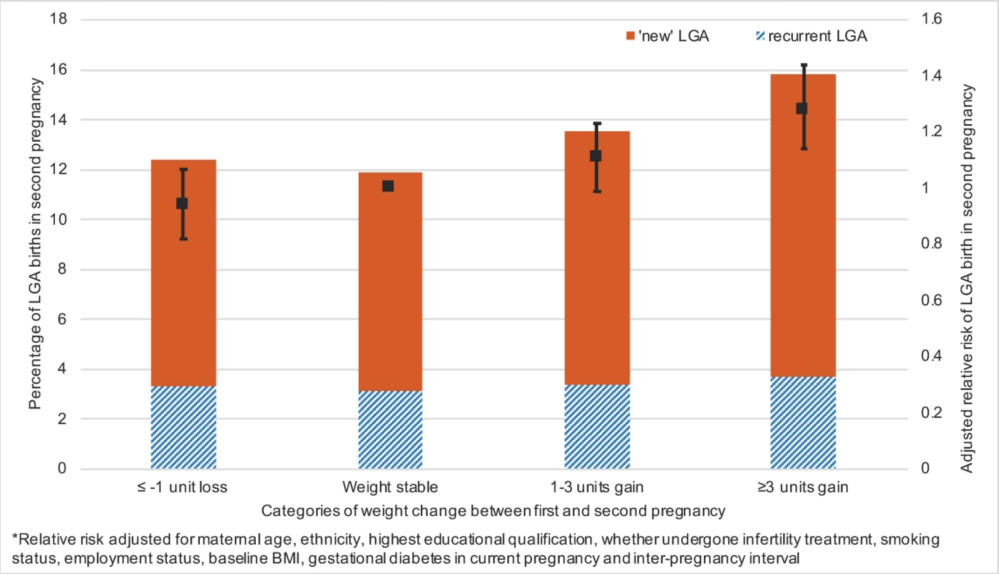

Figure 1: The percentage and risk of LGA births in second pregnancy stratified by maternal inter-pregnancy weight change categories

222x128mm (300 x 300 DPI)

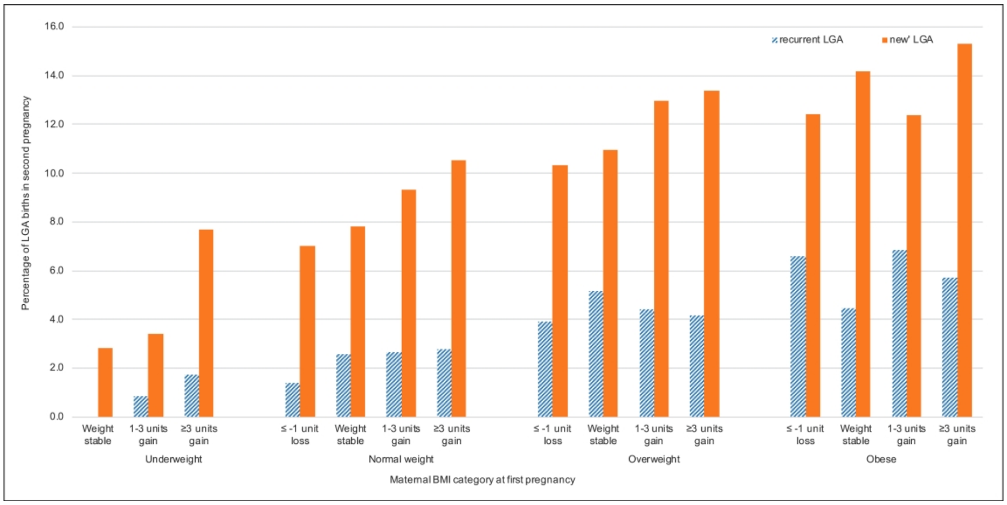

Figure 2: The absolute percentage of LGA births in second pregnancy by inter-pregnancy change in maternal body mass (BMI) stratified by maternal BMI category in the first pregnancy and previous outcome of LGA

346x171mm (300 x 300 DPI)

Reporting checklist for cohort study.

Based on the STROBE cohort guidelines.

Instructions to authors

Complete this checklist by entering the page numbers from your manuscript where readers will find each of the items listed below.

Your article may not currently address all the items on the checklist. Please modify your text to include the missing information. If you are certain that an item does not apply, please write "n/a" and provide a short explanation.

Upload your completed checklist as an extra file when you submit to a journal.

In your methods section, say that you used the STROBE cohort reporting guidelines, and cite them as:

von Elm E, Altman DG, Egger M, Pocock SJ, Gotzsche PC, Vandenbroucke JP. The Strengthening the Reporting of Observational Studies in Epidemiology (STROBE) Statement: guidelines for reporting observational studies.

		Reporting Item	Page Number
Title	#1a	Indicate the study's design with a commonly used term in the title or the abstract	1 and 2
Abstract	#1b	Provide in the abstract an informative and balanced summary of what was done and what was found	2
Background / rationale	#2	Explain the scientific background and rationale for the investigation being reported	3-4
Objectives	#3	State specific objectives, including any prespecified hypotheses	4
Study design	#4	Present key elements of study design early in the paper	4
Setting	#5	Describe the setting, locations, and relevant dates, including periods of recruitment, exposure, follow-up, and data collection	4
Eligibility criteria	#6a	Give the eligibility criteria, and the sources and methods of selection of participants. Describe methods of follow-up.	4

For peer review only

	#6b	For matched studies, give matching criteria and number of exposed and unexposed	n/a
Variables	#7	Clearly define all outcomes, exposures, predictors, potential confounders, and effect modifiers. Give diagnostic criteria, if applicable	4-5
Data sources / measurement	#8	For each variable of interest give sources of data and details of methods of assessment (measurement). Describe comparability of assessment methods if there is more than one group. Give information separately for for exposed and unexposed groups if applicable.	4-5
Bias	#9	Describe any efforts to address potential sources of bias	n/a
Study size	#10	Explain how the study size was arrived at	4
Quantitative variables	#11	Explain how quantitative variables were handled in the analyses. If applicable, describe which groupings were chosen, and why	4-5
Statistical methods	#12a	Describe all statistical methods, including those used to control for confounding	5
	#12b	Describe any methods used to examine subgroups and interactions	5
	#12c	Explain how missing data were addressed	n/a
	#12d	If applicable, explain how loss to follow-up was addressed	n/a
	#12e	Describe any sensitivity analyses	n/a
Participants	#13a	Report numbers of individuals at each stage of study—eg numbers potentially eligible, examined for eligibility, confirmed eligible, included in the study, completing follow-up, and analysed. Give information separately for for exposed and unexposed groups if applicable.	5
	#13b	Give reasons for non-participation at each stage	n/a
	#13c	Consider use of a flow diagram	n/a
Descriptive data	#14a	Give characteristics of study participants (eg demographic, clinical, social) and information on exposures and potential	5-6

For peer review only

		confounders. Give information separately for exposed and unexposed groups if applicable.	
	#14b	Indicate number of participants with missing data for each variable of interest	13-14
	#14c	Summarise follow-up time (eg, average and total amount)	n/a
Outcome data	#15	Report numbers of outcome events or summary measures over time. Give information separately for exposed and unexposed groups if applicable.	5-6, 13-14
Main results	#16a	Give unadjusted estimates and, if applicable, confounder-adjusted estimates and their precision (eg, 95% confidence interval). Make clear which confounders were adjusted for and why they were included	6-7, 15-21
	#16b	Report category boundaries when continuous variables were categorized	4-5, 13-14
	#16c	If relevant, consider translating estimates of relative risk into absolute risk for a meaningful time period	n/a
Other analyses	#17	Report other analyses done—e.g., analyses of subgroups and interactions, and sensitivity analyses	n/a
Key results	#18	Summarise key results with reference to study objectives	7
Limitations	#19	Discuss limitations of the study, taking into account sources of potential bias or imprecision. Discuss both direction and magnitude of any potential bias.	8-9
Interpretation	#20	Give a cautious overall interpretation considering objectives, limitations, multiplicity of analyses, results from similar studies, and other relevant evidence.	9
Generalisability	#21	Discuss the generalisability (external validity) of the study results	8-9
Funding	#22	Give the source of funding and the role of the funders for the present study and, if applicable, for the original study on which the present article is based	10

The STROBE checklist is distributed under the terms of the Creative Commons Attribution License CC-BY. This checklist was completed on 22. August 2018 using http://www.goodreports.org/, a tool made by the EQUATOR Network in collaboration with Penelope.ai

For peer review only

BMJ Open

Is maternal weight gain between pregnancies associated with risk of large-for-gestational age birth? Analysis of a UK population-based cohort

Journal:	*BMJ Open*
Manuscript ID	bmjopen-2018-026220.R2
Article Type:	Research
Date Submitted by the Author:	22-Feb-2019
Complete List of Authors:	Ziauddeen, Nida; University of Southampton, School of Primary Care and Population Sciences, Faculty of Medicine<br>Wilding, Sam; University of Southampton, School of Primary Care and Population Sciences, Faculty of Medicine<br>Roderick, Paul; University of Southampton, School of Primary Care and Population Sciences, Faculty of Medicine<br>Macklon, Nicholas; University of Copenhagen, Zealand University Hospital, Department of Obstetrics and Gynaecology; London Women's Clinic<br>Alwan, Nisreen; University of Southampton, School of Primary Care and Population Sciences, Faculty of Medicine; University of Southampton and University Hospital Southampton NHS Foundation Trust, NIHR Southampton Biomedical Research Centre
**Primary Subject Heading**:	Epidemiology
Secondary Subject Heading:	Epidemiology, Public health
Keywords:	obesity, pregnancy, pregnancy outcome, weight gain, large-for-gestational age

SCHOLARONE™
Manuscripts

Is maternal weight gain between pregnancies associated with risk of large-for-gestational age birth? Analysis of a UK population-based cohort

Nida Ziauddeen[1], Sam Wilding[1], Paul J. Roderick[1], Nicholas S. Macklon[2,3], Nisreen A. Alwan[1,4]

[1] School of Primary Care and Population Sciences, Faculty of Medicine, University of Southampton, Southampton, UK

[2] Department of Obstetrics and Gynaecology, University of Copenhagen, Zealand University Hospital, Roskilde 4000, Denmark

[3] London Women's Clinic, 113-115 Harley Street, London W1G 6AP

[4] NIHR Southampton Biomedical Research Centre, University of Southampton and University Hospital Southampton NHS Foundation Trust, Southampton SO16 6YD, UK

Author emails: N.Ziauddeen@soton.ac.uk, S.A.Wilding@soton.ac.uk, pjr@soton.ac.uk, nism@regionsjaelland.dk, N.A.Alwan@soton.ac.uk

Corresponding author: Nida Ziauddeen

Academic Unit of Primary Care and Population Sciences, Faculty of Medicine, Mailpoint 805, Level C (AC22), South Academic Block, Southampton General Hospital, Tremona Road, Southampton SO16 6YD, UK

Email: N.Ziauddeen@soton.ac.uk

Telephone number: 023 81 206287

Keywords: obesity, pregnancy, pregnancy outcome, weight gain, birth weight

Word count: 4287 words

For peer review only

Abstract

Objective: Maternal overweight and obesity during pregnancy increases the risk of large-for-gestational age (LGA) birth and childhood obesity. We aimed to investigate the association between maternal weight change between subsequent pregnancies and risk of having a LGA birth.

Design: Population-based cohort.

Setting: Routinely collected antenatal healthcare data between January 2003 and September 2017 at University Hospital Southampton, England.

Participants: Health records of women with their first two consecutive singleton live-birth pregnancies were analysed (n=15940).

Primary outcome measure: Risk of LGA, recurrent LGA and 'new' LGA births in the second pregnancy.

Results: Of the 15940 women, 16.0% lost and 47.7% gained weight (≥1 kg/m^2) between pregnancies. A lower proportion of babies born to women who lost ≥1 kg/m^2 (12.4%) and remained weight stable between -1 to 1 kg/m^2 (11.9%) between pregnancies were LGA compared to 13.5% and 15.9% in women who gained 1-3 and ≥3 kg/m^2 respectively. The highest proportion was in obese women who gained ≥3 kg/m^2 (21.2%). Overweight women had a reduced risk of recurrent LGA in the second pregnancy if they lost ≥1 kg/m^2 (adjusted relative risk (aRR) 0.69, 95% CI 0.48-0.97) whereas overweight women who gained ≥3 kg/m^2 were at increased risk of 'new' LGA after having a non-LGA birth in their first pregnancy (aRR 1.35, 95% CI 1.05-1.75). Normal-weight women who gained weight were also at increased risk of 'new' LGA in the second pregnancy (aRR 1.26, 95% CI 1.06-1.50 with gain of 1-3 kg/m^2 and aRR 1.34, 95% CI 1.09-1.65 with gain of ≥3 kg/m^2).

Conclusions: Losing weight after an LGA birth was associated with a reduced LGA risk in the next pregnancy in overweight women, while inter-pregnancy weight gain was associated with an increased 'new' LGA risk. Preventing weight gain between pregnancies is an important prevention measure to achieve better maternal and offspring outcomes.

Article summary

Strengths and limitations of this study

- Utilises antenatal care and birth data from a large population-based cohort including women from all socioeconomic backgrounds
- Objective measurement of both exposure (maternal weight) and outcome in two pregnancies per woman
- Self-reported data for covariates
- Lack of information on breastfeeding duration and maternal weight gain during pregnancy

For peer review only - http://bmjopen.bmj.com/site/about/guidelines.xhtml

Introduction

The prevalence of maternal obesity has been rising over time. It has more than doubled in England between 1989 and 2007 (7.6% to 15.6%), with the proportion of normal weight pregnancies showing a 12% decrease from 65.6% to 53.6%[1]. Maternal overweight and obesity is a key risk factor for adverse maternal and birth outcomes. It also increases the risk of long-term health problems in the child including obesity, cardiovascular disease, diabetes and cognitive and behavioural disorders[2]. Birthweight is a key early life predictor of long-term health outcomes such as obesity and cardiovascular disease[3] and potentially acts as a mediator on the causal pathway between maternal obesity and long-term offspring outcomes. The incidence of large-for-gestational age (LGA) birth, defined as >90th percentile weight for gestational age, has increased over time in high-income countries[4,5]. LGA is associated with both childhood[6,7] and adult obesity[8-10]. A key risk factor for LGA birth is gestational diabetes (GDM)[11], the incidence of which has also increased over time[12,13]. Offspring of mothers with gestational diabetes have increased risk of childhood overweight and obesity[14,15]. Maternal obesity is an established risk factor for both GDM and LGA birth[16]. Change in maternal body mass index (BMI) between pregnancies could modify the risk of LGA birth in the subsequent pregnancy.

Birthweight, on average, increases with parity. First-born infants tend to have the lowest birthweight among their younger siblings[17-19] up to the fourth pregnancy[20]. However, birthweight was found to decrease with parity for women who had short intervals between their pregnancies (<12 months) while the increase in birthweight with parity was more pronounced in women with long intervals (>24 months)[20]. Also, maternal weight change between pregnancies was found to modify the relationship between parity and birthweight. Women who returned to their pre-pregnancy weight before the next conception had infants who weighed less than infants of women who retained or gained weight between pregnancies[20]. In a UK- based study, women who lost at least six kilograms between their first and second pregnancy had a smaller average increase in birthweight of the second baby compared to women who gained ten kilograms or more (in a 1.60m tall woman, 6 kg equates to approximately 2.3 kg/m^2 and 10 kg to approximately 3.8 kg/m^2)[18].

A large US study showed that women were at an increased risk of having an LGA baby in the second pregnancy if their pre-pregnancy BMI category increased towards overweight or obese between their first and second pregnancies. This applied to all first pregnancy BMI categories, except underweight women who became normal weight by the start of their second pregnancy. Overweight and obese women who dropped BMI category by their second pregnancy remained at an increased risk of LGA birth, but had a lower risk compared to women whose BMI category increased between pregnancies[21].

Another US-based study showed that inter-pregnancy weight gain of ≥2 kg/m^2 in obese women was associated with increased risk of LGA. Weight loss of ≥2 kg/m^2 was associated with a lower adjusted LGA risk compared to the women who maintained their weight within 2 kg/m^2 change between pregnancies[22].

Two studies found a reduced risk of 'new' LGA in the second pregnancy following a non-LGA birth in the first pregnancy with inter-pregnancy weight loss of >1 kg/m^2, and an increased risk with modest (1-3 kg/m^2) and large (≥3 kg/m^2) weight gain. In stratified analysis, the association was stronger in women with a first pregnancy BMI of <25 kg/m^2 [23,24]. A third study only found an increased risk of 'new' LGA in normal weight women who gained ≥4 kg/m^2 between pregnancies and no association in overweight women[25].

To our knowledge, only one study has examined the risk of recurrent LGA (occurring in both first and second pregnancies) in relation to maternal weight change between pregnancies [26]. The study, conducted in Aberdeen, Scotland, included 24520 women of which 813 women had LGA births in both pregnancies. Inter-pregnancy weight gain (≥2 kg/m^2) was associated

For peer review only - http://bmjopen.bmj.com/site/about/guidelines.xhtml

with increased risk of recurrent LGA, while weight loss (≥2 kg/m^2) was protective. Women with BMI <25kg/m^2 were at increased risk of recurrent LGA on gaining weight whereas women with BMI ≥25kg/m^2 were at reduced risk of recurrent LGA on losing weight[26].

In this study, we aimed to investigate the association between the incidence of LGA, recurrent LGA and 'new' LGA births in the second pregnancy and maternal change in BMI between the first and second pregnancies, stratifying by maternal BMI category in the first pregnancy, in a population-based cohort in the South of England.

Methods

This is a population-based cohort of prospectively collected routine healthcare data for antenatal care between January 2003 and September 2017 at University Hospital Southampton, Hampshire, UK. This included all women delivering at this hospital (n= 82098 pregnancies), which is a regional centre for maternity care in and around Southampton. Records of women with their first two consecutive singleton live birth pregnancies were included. Records with unfeasible weight (<30kg), height (>2m) and gestational age (>301 days) values were excluded.

Exposure assessment

Maternal weight in kilograms was routinely measured by a midwife at the first antenatal (booking) appointment of each pregnancy, which is recommended to take place ideally by 10 weeks gestation in the UK, according to the National Institute for Health and Care Excellence Guidelines [27]. Any woman who had a booking appointment at or after 24 weeks of pregnancy was excluded. Height was self-reported. BMI was calculated as weight (in kg) divided by height (in metres) squared.

BMI at the start of the first pregnancy was categorised as underweight (BMI <18.5 kg/m^2), normal weight (18.5 to 24.9 kg/m^2), overweight (25.0 to 29.9 kg/m^2) and obese (≥30 kg/m^2). Change in BMI was calculated as the difference in BMI measured at the booking appointments of the first two consecutive live birth pregnancies for each woman. This change in BMI was then categorised as weight loss (≥1 kg/m^2), weight stable (-1 to 1 kg/m^2) and two categories of weight gain (1- 3 kg/m^2 and ≥3 kg/m^2).

Outcome assessment

Birthweight (grams) was measured by healthcare professionals at birth as part of routine care. Gestational age was based on a dating ultrasound scan which routinely takes place between 10 and 13 weeks gestation[27]. Age- and gender- specific birthweight centiles were calculated using reference values for England and Wales provided in the most recently released national data[28]. Large-for-gestational age was defined as >90th percentile weight for gestational age. This was only defined for babies born between 24 to 42 weeks gestation as reference values only exist for these gestational ages and with determinate gender.

Covariates

Maternal date of birth is recorded at the booking appointment and converted to age (in years) on extraction of the dataset to maintain anonymity. Highest maternal educational

For peer review only - http://bmjopen.bmj.com/site/about/guidelines.xhtml

qualification was self-reported and categorised as primary, secondary, college, undergraduate, postgraduate, graduate and none. For the purposes of this analysis, this was condensed to three categories - secondary (GCSE) and under, college (A levels) and university degree or above. Self-reported ethnicity was recorded under 16 categories and condensed to White, Mixed, Asian, Black/African/Caribbean and Other. Categories of not asked and not stated were coded as missing. Smoking was self-reported as current smoking or non-smoking. Non-smokers were further asked if they had ever smoked or had previously smoked and quit. This was categorised as stopped more than 12 months before conception, stopped less than 12 months before conception or stopped when pregnancy confirmed. Employment status was self-reported at booking appointment and categorised as employed, unemployed, in education, and not specified. Infertility treatment was categorised as no/investigations only and yes (hormonal only, in-vitro fertilisation, gamete intrafallopian transfer and other surgical) in either one or both pregnancies. In this population, an oral glucose tolerance test was used for screening for GDM in women with one or more risk factors (BMI > 30kg/m^2; GDM in previous pregnancy; previous baby weighing $\geq$4.5kg; diabetes in parents or siblings and of Asian, African-Caribbean or Middle Eastern ethnicity)[29]. GDM diagnosis was then reported in the database. Inter-pregnancy interval was defined as the interval between the first live birth and conception of the second pregnancy. The difference in days between two consecutive live births was calculated and gestational age of the latter birth subtracted from this to derive the inter-pregnancy interval.

Statistical analysis

All analysis was performed using Stata 15[30]. Univariable comparisons were carried out using ANOVA for continuous variables and chi square test for categorical variables. Generalised linear regression with log link[31] was used to examine the association between the categorised variable of maternal change in BMI between pregnancies with risk of LGA in the second pregnancy. This was analysed first in the whole sample and then stratified by 'baseline' maternal BMI category as calculated in the first antenatal appointment of the first pregnancy.

Risk of LGA in the second pregnancy was explored in the whole sample adjusting for previous pregnancy outcome of LGA. The risk of 'new' LGA in second pregnancy after having a non-LGA baby in the first pregnancy was explored in the sub-sample of women who had non-LGA births in the first pregnancy. The risk of recurrent LGA (LGA in both pregnancies) was explored in a sub-sample of women who had LGA births in the first pregnancy.

Initial univariable analysis was followed by multivariable models adjusting for potential confounding factors – maternal age, ethnicity, highest educational qualification, whether or not undergone infertility treatment, employment status, smoking behaviour in second pregnancy, baseline BMI, GDM in second pregnancy and inter-pregnancy interval. Sensitivity analysis was conducted adding gestational age at booking in the second pregnancy to the models.

A statistical significance level of 0.05 with 95% confidence intervals was used in the regression models.

Ethical considerations

All data were fully anonymised by the data holder before being accessed by the research team. Ethics approval was granted by the University of Southampton Faculty of Medicine Ethics Committee: study ID 25508.

For peer review only - http://bmjopen.bmj.com/site/about/guidelines.xhtml

Patient and Public Involvement

Patients and public were not involved in setting the research question or the outcome measures, nor were they involved in developing plans for the design or implementation of the study. However, pregnant woman and mothers of young children have been involved in the planning stages of a research project building on this analysis.

Results

The first and second pregnancies of 15940 women were included. Of these, 16.0% of women lost ≥1 kg/m^2, 36.3% remained weight stable (-1 to 1 kg/m^2), 27.9% gained 1-3 kg/m^2 and 19.8% gained ≥3 kg/m^2 between their first and second live birth pregnancies. Weight loss of >2 kg/m^2 was observed in 7.3% of women whereas 30.5% gained >2 kg/m^2. Mean BMI at second pregnancy booking was 30.8 kg/m^2 (standard deviation (SD) 5.9) in women who gained ≥3 kg/m^2, 25.9 kg/m^2 (SD 4.7) in women who gained 1-3kg/m^2 , 24.1 kg/m^2 (SD 5.1) in women who lost weight, and 23.8 kg/m^2 (SD 4.4) women whose weight remained stable between pregnancies ($p<0.001$) (Table 1).

Women who gained ≥3 kg/m^2 by the start of their second pregnancy were more likely to be smokers, unemployed, with lower educational attainment and to have a longer inter-pregnancy interval, compared to those who maintained a stable weight between pregnancies. Mean maternal age was lowest in the women who gained ≥3 kg/m^2 (27.3 years, SD 5.5) and highest in the women who remained weight stable (29.8 years, SD 5.3). Mean maternal age in women who lost weight was 28.7 years (SD 5.4).

Mothers who gained ≥3 kg/m^2 were more likely to be obese (48.3%) at the start of the second pregnancy compared to 16.1% in women who gained 1-3 kg/m^2, 9.2% in women who remained weight stable and 11.9% in women who lost ≤1 kg/m^2.

Figure 1 shows the percentage of women in each BMI category in the first and second pregnancy and the weight gain over time. There has been a decline in normal weight women at first pregnancy and a slight increase in overweight and obese women over time. There also was a slight decline in the percentage of women gaining ≥3 kg/m^2 and a slight increase in those gaining 1-3 kg/m^2.

The proportion of LGA births were higher in all BMI categories in the second pregnancy (Figure 2). A lower proportion of babies born to women who lost weight (12.4%) or remained weight stable (11.9%) between pregnancies were LGA compared to 13.5% in women who gained 1-3 kg/m^2 and 15.9% in women who gained ≥3 kg/m^2 ($p<0.001$) (Table 1, Figure 3). Compared to normal weight women, overweight and obese women were at increased risk of LGA births in both pregnancies with risk highest in obese women (unadjusted relative risk (RR) 2.06, 95% CI 1.78 to 2.38 and 1.86, 95% CI 1.69 to 2.05 in first and second pregnancy respectively). The lowest proportion of LGA births in the second pregnancy was in underweight women in the first pregnancy who remained weight stable (2.8%), while the highest was in obese women who gained ≥3 kg/m^2 (21.2%). Within BMI categories, recurrent LGA was lowest in normal weight and overweight women who lost weight and highest in obese women who gained 1-3 kg/m^2.

Women who gained ≥3 kg/m^2 were at increased risk of LGA in the second pregnancy in the full sample compared to remaining weight stable (aRR 1.28, 95% CI 1.14 to 1.44) (Figure 3).

For peer review only - http://bmjopen.bmj.com/site/about/guidelines.xhtml

There was a significantly reduced risk of recurrent LGA birth in the second pregnancy in overweight women who had a LGA infant in the first pregnancy and lost ≥1 kg/m^2 in weight (aRR 0.69, 95% CI 0.48 to 0.97) (Table 2, supplementary Figure 1). No association was observed between risk of recurrent LGA and maternal BMI change between pregnancies in underweight, normal weight and obese women.

There was an increased risk of 'new' LGA birth in the second pregnancy after having a non-LGA infant in the first pregnancy in normal weight women who gained 1-3 kg/m^2 (aRR 1.26, 95% CI 1.06 to 1.50) and in normal weight and overweight women who had gained ≥3 kg/m^2 weight (aRR 1.34, 95% CI 1.09 to 1.65, aRR 1.35, 95% CI 1.05 to 1.75, respectively) (Table 3, supplementary Figure 2). No association was observed between the risk of 'new' LGA in the second pregnancy and maternal BMI inter-pregnancy change in obese women.

Discussion

This study examined the association between change in women's BMI between their first and second live birth pregnancies and risk of LGA birth in the second pregnancy in a population-based cohort of 15940 women in the South of England. Almost half of the sample (48%) of women gained ≥1 kg/m^2 in the time between the first antenatal care visits during their first and second pregnancies. The proportion of LGA births was significantly higher in women with an inter-pregnancy weight gain of ≥3 kg/m^2 (16%) compared to women who lost weight (12%) and those who remained weight stable (12%) between pregnancies. Overweight women who lost ≥1 kg/m^2 had a reduced risk of recurrent LGA. Normal weight women who gained 1–3 kg/m^2 and both normal weight and overweight women who gained ≥3 kg/m^2 between pregnancies had an increased risk of LGA birth in their second pregnancy after a non-LGA birth in the first.

Compared to the population-based Swedish cohort which carried out a similar analysis for LGA and other outcomes in 151025 women using data from 1992 to 2001, a lower proportion of women remained weight stable in our cohort (46% compared to 36%) and a higher proportion lost (11% compared to 16%) or gained (43% compared to 48%) weight. Amongst women who gained weight, a higher proportion gained ≥3 kg/m^2 in this cohort (20%) compared to the Swedish cohort (11%)[23]. Similarly, in comparison to a population-based cohort of 24520 women in Aberdeen, Scotland; for the period 1986 to 2013, a larger proportion of women in our study both lost and gained weight[26]. The differences could reflect the increase in the prevalence of maternal overweight and obesity over time since our data are more recent.

In the adjusted model utilising the full sample, we showed an increased risk of LGA in the second pregnancy for inter-pregnancy weight gain compared to weight remaining stable. In a population-based cohort in the US, women were found to be at increased risk of LGA in the second pregnancy if their pre-pregnancy BMI category changed towards overweight or obese from first to second pregnancy regardless of their BMI category in first pregnancy except in underweight women who increased to normal weight[21]. This study is different to ours in that it only examined risk in second pregnancy without adjustment for LGA outcome in first pregnancy. It also considered weight change as change in BMI category only, while we studied change in maternal BMI regardless of whether BMI category has changed or not in the second pregnancy.

In obese women in the US, inter-pregnancy weight gain of ≥2 kg/m^2 was associated with increased risk of LGA and a weight loss of ≥2 kg/m^2 was associated with

For peer review only - http://bmjopen.bmj.com/site/about/guidelines.xhtml

decreased risk compared to the reference group of weight maintained (between >-2 and <2 kg/m^2)[22]. We found no association between weight change and risk of second pregnancy LGA in women who were obese at the start of their first pregnancy. This may be because obese women are already at increased risk of LGA births, and the average inter-pregnancy BMI change in this subgroup was not large enough to detect a further increase in risk. Greater efforts are needed for primary prevention of obesity in women of child bearing age and obese women need more effective weight loss strategies in inter-partum period to assess impact on LGA and other outcomes.

Risk of recurrent LGA was analysed in one previous study in Scotland which found that inter-pregnancy weight gain (≥2 kg/m^2) was associated with increased risk of recurrent LGA. In that study, weight loss (≥2 kg/m^2) was associated with reduced LGA risk. Stratification by first pregnancy BMI showed that women with BMI <25kg/m^2) were at increased risk of recurrent LGA on gaining ≥2 kg/m^2 whereas women with BMI ≥25kg/m^2 were at reduced risk of recurrent LGA on losing ≥2 kg/m^2 weight[26]. We showed a similar reduction in risk in overweight women who lost ≥1 BMI unit between pregnancies, but found no association in normal weight women. This difference in findings may be because the <25kg/m^2 group in the previous Scottish study included underweight women whereas our stratified analysis examined normal weight women separately to underweight women.

We showed an increased risk of 'new' LGA in the second pregnancy (after a non-LGA birth in the first pregnancy) with inter-pregnancy weight gain compared to remaining weight stable. After stratification by BMI, we found that this association between inter-pregnancy weight gain and new LGA remained only in normal-weight and overweight women. The findings from this study are in line with findings with other studies in Scotland[24] and Sweden[23] which found increased risk of 'new' LGA with modest (1-3 kg/m^2) and large (≥3 kg/m^2) weight gain. Both studies also found a decreased risk with inter-pregnancy weight loss of >1 kg/m^2 which was not found in our study. Both studies stratified BMI as < and ≥25kg/m^2, while we further stratified the ≥25kg/m^2 category as overweight (BMI 25-29.9kg/m^2) and obese (≥30kg/m^2) and found an increased risk of 'new' LGA in overweight, but not in obese women. We carried out sensitivity analysis merging overweight and obese categories and found increased risk in this category (data not shown) suggesting that the results are comparable to previous studies.

Women included in this analysis had a range of inter-pregnancy interval of less than 1 to up to 12 years and thus weight change could be due to postpartum weight retention or late postpartum weight gain. There is evidence that women who do not lose pregnancy weight at one year postpartum are more likely to retain weight longer term[32]. We examined the risk of maternal inter-pregnancy weight gain with length of the inter-pregnancy interval and found that women with an interval of 12-23 months were least likely to start the next pregnancy at a higher weight[33]. We also examined the length of the inter-pregnancy interval as a predictor for LGA risk adjusting for inter-pregnancy weight change and found no association[34].

The Development Origins of Health and Disease concept suggests that adverse exposures during development could lead to enhanced susceptibility in the foetus thus increasing the risk of non-communicable diseases in later life. Although the focus has previously been on exposures during pregnancy, the importance of the preconception period is now recognised[35-37]. Efforts to systematically identify women in the preconception period to improve health and lifestyle during conception are underway[37]. Promoting health of all women of child-bearing age with targeting of women and partners planning a pregnancy has been identified as an effective approach to improving preconception health[36]. It is difficult to identify all women who are planning a pregnancy but as the inter-conception period is also the preconception period for the next pregnancy, it is important to engage with women during this period to optimise their and their children's health.

For peer review only - http://bmjopen.bmj.com/site/about/guidelines.xhtml

Future research that characterises the predictors of postpartum weight change would help design interventions to support postpartum weight loss and prevent weight gain. Key to this is an understanding of the pattern of weight change during this period as well as identifying the optimal setting and delivery of the intervention. Support with healthy eating and physical activity is more commonly received during pregnancy than after birth. Even when lifestyle advice is received postpartum, it was found not to be associated with healthy diet or physical activity behaviours[38]. Most interventions that have been successful in limiting and promoting postpartum weight loss were combined diet and physical activity interventions with self-monitoring[39]. However, the timing of engaging women and length of intervention or engagement are important with one study showing that an intervention from 16 weeks pregnancy to six months postpartum was more effective than the same intervention from birth to six months postpartum intervention[40].

As pregnancy and early postpartum is a period of major change for women and their families, interventions need to be carefully designed to be attractive, flexible, affordable and feasible for women at this stage with competing priorities and time demands. Focus during the postpartum period in the UK healthcare system is mostly on child health and development. The feasibility and effectiveness of better utilising contact time with health professionals during the two years after birth to engage and support maternal health needs to be explored. There may also be a role for mutual support groups for mothers. There is additionally a need to recognise that weight management issues are greater in more disadvantaged mothers so there is also the issue of identifying the most effective weight management strategies for such mothers to reduce social inequity in subsequent birth and maternal outcomes. Weight gain does not occur in isolation and usually combined with other risk factors particularly in socioeconomically disadvantaged groups and hence a holistic approach taking into account priority setting for these families should be considered.

Strengths and limitations

This is a relatively large population-based cohort including women from all socioeconomic and ethnic backgrounds delivering at a large maternity centre in Southampton, UK, thus representative of the regional population. According to the UK Department of Communities and Local Government English indices of deprivation report, Southampton is more deprived than average with the situation having worsened between 2010 and 2015[41]. However, about half of the women included in this analysis reside in the rest of Hampshire (the region where Southampton is situated), which is less deprived. Our sample was 87% of White ethnicity, which is comparable to the 2011 England and Wales population census of 86% White[42]. The analysis was adjusted for several key confounders that were reasonably complete (96% complete for ethnicity and employment status). Both the maternal weight (used to calculate exposure) and birthweight in this study were objectively measured by healthcare professionals as part of routine antenatal and delivery care.

An important limitation was the lack of information on gestational weight gain during pregnancy, breastfeeding duration/exclusivity and paternal characteristics/behaviour, which are potential confounders in the association between maternal inter-pregnancy weight gain and LGA birth[43]. We adjusted for if first feed was breast milk as a proxy for breastfeeding initiation in sensitivity analysis and the results remained unchanged (not shown). Women who had their first booking appointment later into the pregnancy (more than 24 weeks) were excluded from the analysis in order to ensure comparability of weight measurements between pregnancies. We also adjusted for gestational age at booking, as this was the point when maternal BMI was measured, in sensitivity analysis and the estimates remained similar. Some of the confounding factors which were accounted for in the analysis were self-reported, however the information was collected prospectively, therefore any measurement error in likely to be non-differential. Another limitation is that these findings are based on observational data so inferences about causation cannot be drawn and the risk of residual confounding influencing the results needs to be considered.

For peer review only - http://bmjopen.bmj.com/site/about/guidelines.xhtml

In conclusion, maternal weight gain of 1 or more kg/m^2 between first and second pregnancy had a prevalence of 48%, and it was associated with risk of LGA in the second pregnancy in this English cohort. Risk of 'new' LGA was higher in normal weight and overweight women who gained weight after a non-LGA birth in their first pregnancy compared to those who remained weight stable. Overweight women were at a lower risk of a recurrent LGA birth in their second pregnancy if they lost weight between pregnancies. Greater efforts are needed for primary prevention of overweight and obesity in women of child bearing age. Supporting efforts to lose weight in overweight and obese women between pregnancies, and stop weight gain in all women planning to have further children (except those who are underweight) are important preventive measures of subsequent adverse maternal and offspring health outcomes.

Author Contributions

Study design (NZ, PJR, NSM, NAA), data analysis (NZ, SW), acquisition and interpretation of the data (NZ, NAA), drafting of the manuscript (NZ), revising for content (NZ, SW, PJR, NSM, NAA) and approval of final version before submission (NZ, SW, PJR, NSM, NAA). NAA is the project's PI.

Data statement

The data owner is University Hospital Southampton NHS Trust. Anonymised data are only available upon request from the PI (NAA) conditional on approval of the appropriate institutional ethics and research governance processes.

Acknowledgements

We would like to thank David Cable (Electronic Patient Records Implementation and Service Manager) and Florina Borca (Senior Information Analyst R&D) at University Hospital Southampton for support in accessing the data used in this study. We also thank clinical colleagues at the Princess Anne Hospital, Southampton for collecting the data.

Funding

This work is supported by a University of Southampton Primary Care and Population Sciences PhD studentship (to NZ), and an Academy of Medical Sciences and Wellcome Trust Grant [AMS_HOP001\1060 to NAA]. NAA is also in receipt of research support from the National Institute for Health Research through the NIHR Southampton Biomedical Research Centre.

Competing interests

The authors have no competing interests to declare.

References

For peer review only - http://bmjopen.bmj.com/site/about/guidelines.xhtml

1. Heslehurst N, Rankin J, Wilkinson JR, et al. A nationally representative study of maternal obesity in England, UK: trends in incidence and demographic inequalities in 619 323 births, 1989-2007. *Int J Obes (Lond)* 2010;34(3):420-8.
2. Poston L. Maternal obesity, gestational weight gain and diet as determinants of offspring long term health. *Best Pract Res Clin Endocrinol Metab* 2012;26(5):627-39.
3. Barker DJP. Fetal origins of coronary heart disease. *BMJ* 1995;311:171-74.
4. Kramer MS, Morin I, Yang H, et al. Why are babies getting bigger? Temporal trends in fetal growth and its determinants. *J Pediatr* 2002;141(4):538-42.
5. Surkan PJ, Hsieh CC, Johansson AL, et al. Reasons for increasing trends in large for gestational age births. *Obstet Gynecol* 2004;104(4):720-6.
6. Reilly JJ, Armstrong J, Dorosty AR, et al. Early life risk factors for obesity in childhood: cohort study. *BMJ* 2005;330(7504):1357.
7. Danielzik S, Czerwinski-Mast M, Langnase K, et al. Parental overweight, socioeconomic status and high birth weight are the major determinants of overweight and obesity in 5-7 y-old children: baseline data of the Kiel Obesity Prevention Study (KOPS). *Int J Obes Relat Metab Disord* 2004;28(11):1494-502.
8. Curhan GC, Willett WC, Rimm EB, et al. Birth Weight and Adult Hypertension, Diabetes Mellitus, and Obesity in US Men. *Circulation* 1996;94:3246-50.
9. Curhan GC, Chertow GM, Willett WC, et al. Birth Weight and Adult Hypertension and Obesity in Women. *Circulation* 1996;94:1310-15.
10. Phillips DIW, Young JB. Birth weight, climate at birth and the risk of obesity in adult life. *International Journal of Obesity* 2000;24:281-87.
11. Casey BM, Lucas MJ, McIntire DD, et al. Pregnancy Outcomes in Women With Gestational Diabetes Compared With the General Obstetric Population. *Obstetrics & Gynecology* 1997;90(6):869-73.
12. Hunt KJ, Schuller KL. The Increasing Prevalence of Diabetes in Pregnancy. *Obstet Gynecol Clin North Am.* 2007;34(2):173-99.
13. Ignell C, Claesson R, Anderberg E, et al. Trends in the prevalence of gestational diabetes mellitus in southern Sweden, 2003-2012. *Acta Obstet Gynecol Scand* 2014;93(4):420-4.
14. Baptiste-Roberts K, Nicholson WK, Wang NY, et al. Gestational diabetes and subsequent growth patterns of offspring: the National Collaborative Perinatal Project. *Matern Child Health J* 2012;16(1):125-32.
15. Zhu Y, Olsen SF, Mendola P, et al. Growth and obesity through the first 7 y of life in association with levels of maternal glycemia during pregnancy: a prospective cohort study. *Am J Clin Nutr* 2016;103(3):794-800.
16. Marchi J, Berg M, Dencker A, et al. Risks associated with obesity in pregnancy, for the mother and baby: a systematic review of reviews. *Obes Rev* 2015;16(8):621-38.
17. Swamy GK, Edwards S, Gelfand A, et al. Maternal age, birth order, and race: differential effects on birthweight. *J Epidemiol Community Health* 2012;66(2):136-42.
18. Wilcox MA, Chang AMZ, Johnson IR. The effects of parity on birthweight using successive pregnancies. *Acta Obstetricia et Gynecologica Scandinavica* 1996;75(5):459-63.
19. Beaty TH, Skjaerven R, Breazeale DR, et al. Analyzing Sibship Correlations in Birth Weight Using Large Sibships From Norway. *Genetic Epidemiology* 1997;14:423-33.
20. Hinkle SN, Albert PS, Mendola P, et al. The association between parity and birthweight in a longitudinal consecutive pregnancy cohort. *Paediatr Perinat Epidemiol* 2014;28(2):106-15.
21. Getahun D, Ananth CV, Peltier MR, et al. Changes in prepregnancy body mass index between the first and second pregnancies and risk of large-for-gestational-age birth. *Am J Obstet Gynecol* 2007;196(6):530 e1-8.
22. Jain AP, Gavard JA, Rice JJ, et al. The impact of interpregnancy weight change on birthweight in obese women. *Am J Obstet Gynecol* 2013;208(3):205 e1-7.
23. Villamor E, Cnattingius S. Interpregnancy weight change and risk of adverse pregnancy outcomes: a population-based study. *Lancet* 2006;368(9542):1164-70.

For peer review only

For peer review only - http://bmjopen.bmj.com/site/about/guidelines.xhtml

24. Wallace JL, Bhattacharya S, Campbell DM, et al. Inter-pregnancy weight change impacts placental weight and is associated with the risk of adverse pregnancy outcomes in the second pregnancy. *BMC Pregnancy and Childbirth* 2014;14(40).
25. McBain RD, Dekker GA, Clifton VL, et al. Impact of inter-pregnancy BMI change on perinatal outcomes: a retrospective cohort study. *Eur J Obstet Gynecol Reprod Biol* 2016;205:98-104.
26. Wallace JM, Bhattacharya S, Campbell DM, et al. Inter-Pregnancy Weight Change and the Risk of Recurrent Pregnancy Complications. *PLoS One* 2016;11(5):e0154812.
27. National Institute for Health Care and Excellence. Antenatal care for uncomplicated pregnancies. Manchester, UK, 2008.
28. Norris T, Seaton SE, Manktelow BN, et al. Updated birth weight centiles for England and Wales. *Archives of Disease in Childhood. Fetal and Neonatal Edition* 2017.
29. National Health Service. *Gestational Diabetes*. https://www.nhs.uk/conditions/gestational-diabetes/ (accessed 26/11/2018).
30. Stata Statistical Software: Release 15 [program]. College Station, TX: StataCorp LLC, 2017.
31. Cummings P. Methods for estimating adjusted risk ratios. *The Stata Journal* 2009;9(2):175-96.
32. Linné Y, Dye L, Barkeling B, et al. Long-Term Weight Development in Women: A 15-Year Follow-up of the Effects of Pregnancy. *Obesity Research* 2004;12(7):1166-78.
33. Ziauddeen N, Roderick PJ, Macklon NS, et al. Length of inter-pregnancy interval and subsequent preconception adiposity: Findings from a population-based cohort in the South of England. *Revue d'Épidémiologie et de Santé Publique* 2018;66:S374.
34. Ziauddeen N, Roderick PJ, Macklon NS, et al. LB5 Is the duration of the preceding inter-pregnancy interval associated with offspring's size at birth? – analysis of a UK population-based cohort. *Journal of Epidemiology and Community Health* 2018;72(Suppl 1):A43.
35. Fleming TP, Watkins AJ, Velazquez MA, et al. Origins of lifetime health around the time of conception: causes and consequences. *The Lancet* 2018;391(10132):P1842-52.
36. Barker M, Dombrowski SU, Colbourn T, et al. Intervention strategies to improve nutrition and health behaviours before conception. *The Lancet* 2018;391(10132):P1853-64.
37. Stephenson J, Heslehurst N, Hall H, et al. Before the beginning: nutrition and lifestyle in the preconception period and its importance for future health. *The Lancet* 2018;391(10132):P1830-41.
38. van der Pligt P, Olander EK, Ball K, et al. Maternal dietary intake and physical activity habits during the postpartum period: associations with clinician advice in a sample of Australian first time mothers. *BMC Pregnancy Childbirth* 2016;16:27.
39. van der Pligt P, Willcox J, Hesketh KD, et al. Systematic review of lifestyle interventions to limit postpartum weight retention: implications for future opportunities to prevent maternal overweight and obesity following childbirth. *Obes Rev* 2013;14(10):792-805.
40. Huang TT, Yeh CY, Tsai YC. A diet and physical activity intervention for preventing weight retention among Taiwanese childbearing women: a randomised controlled trial. *Midwifery* 2011;27(2):257-64.
41. Department for Communities and Local Government. The English Indices of Deprivation 2015, 2015.
42. Office for National Statistics. Ethnicity and National Identity in England and Wales 201, 2012.
43. Nohr EA, Vaeth M, Baker JL, et al. Combined associations of prepregnancy body mass index and gestational weight gain with the outcome of pregnancy. *American Journal of Clinical Nutrition* 2008;87(6):1750-59.

Figure legends

For peer review only - http://bmjopen.bmj.com/site/about/guidelines.xhtml

Figure 1: The percentage of women in each body mass index (BMI) category in the first and second pregnancy and weight gain over time in the cohort (2003-2017)

Figure 2: The percentage of large-for-gestational age (LGA) births in first and second pregnancy by maternal body mass index (BMI) category

Figure 3: The percentage and risk of large-for-gestational age (LGA) births in second pregnancy stratified by maternal inter-pregnancy weight change categories

Supplementary Figure 1: Associations between risk of recurrent large-for-gestational age (LGA) birth in the second pregnancy and change in maternal body mass index (BMI) between pregnancies as measured at the first antenatal visit of each pregnancy stratified by BMI category in the first pregnancy

Supplementary Figure 2: Associations between the risk of 'new' large-for-gestational age (LGA) birth in the second pregnancy following a non-LGA birth in the first pregnancy and change in maternal body mass index (BMI) between pregnancies measured at the first antenatal visit of each pregnancy stratified by BMI category in the first pregnancy

Table legends:

Table 1: Maternal and birth characteristics in the second live birth pregnancy categorised by maternal weight change from the first livebirth pregnancy for the period of January 2003 - September 2017, University Hospital Southampton NHS Foundation Trust, Hampshire, England

Table 2: Associations between risk of recurrent large-for-gestational age (LGA) birth in the second pregnancy and change in maternal body mass index (BMI) between pregnancies as measured at the first antenatal visit of each pregnancy stratified by BMI category in the first pregnancy

Table 3: Associations between the risk of 'new' large-for-gestational age (LGA) birth in the second pregnancy following a non-LGA birth in the first pregnancy and change in maternal body mass index (BMI) between pregnancies measured at the first antenatal visit of each pregnancy stratified by BMI category in the first pregnancy

For peer review only - http://bmjopen.bmj.com/site/about/guidelines.xhtml

Table 1: Maternal and birth characteristics in the second live birth pregnancy categorised by maternal weight change gain from the first live birth pregnancy for the period of January 2003 - September 2017, University Hospital Southampton NHS Foundation Trust, Hampshire, England

	Lost ≤ -1 kg/m^2 from previous pregnancy	Weight stable (>-1 to <1 kg/m^2)	Gained 1-3 kg/m^2 from previous pregnancy	Gained ≥3 kg/m^2 from previous pregnancy	p*
N	2548	5785	4446	3161	
Maternal age, years (mean ± SD)	28.7 ± 5.4	29.8 ± 5.3	29.2 ± 5.4	27.3 ± 5.5	<0.001
Timing of first booking appointment, weeks (mean ± SD)	10.8 ± 2.3	11.0 ± 2.3	11.1 ± 2.4	11.0 ± 2.6	<0.001
Maternal BMI at booking, kg/m^2 (mean ± SD)	24.1 ± 5.1	23.8 ± 4.4	25.9 ± 4.7	30.8 ± 5.9	<0.001
Maternal BMI at booking in first pregnancy (%, 95% CI)					
Underweight (< 18.5)	0.8 (0.5 to 1.2)	4.3 (3.8 to 4.8)	5.3 (4.7 to 6.0)	3.7 (3.1 to 4.4)	<0.001
Normal weight (18.5 to 24.9)	47.6 (45.6 to 49.5)	67.4 (66.2 to 68.6)	62.5 (61.0 to 63.9)	49.0 (47.2 to 50.7)	
Overweight (25.0 to 29.9)	30.1 (28.3 to 31.9)	19.4 (18.4 to 20.5)	22.0 (20.8 to 23.3)	29.5 (28.0 to 31.2)	
Obese (≥30.0)	21.5 (19.9 to 23.2)	8.9 (8.2 to 9.7)	10.2 (9.3 to 11.1)	17.8 (16.5 to 19.2)	
Maternal BMI at booking in second pregnancy (%, 95% CI)					
Underweight (< 18.5)	6.9 (5.9 to 7.9)	4.3 (3.8 to 4.8)	0.6 (0.4 to 0.9)	0.0 (0.0 to 0.2)	<0.001
Normal weight (18.5 to 24.9)	61.1 (59.2 to 63.0)	66.8 (65.6 to 68.1)	50.7 (49.2 to 52.1)	14.9 (13.7 to 16.2)	
Overweight (25.0 to 29.9)	20.1 (18.6 to 21.7)	19.7 (18.7 to 20.7)	32.6 (31.2 to 34.0)	36.7 (35.0 to 38.4)	
Obese (≥30.0)	11.9 (10.7 to 13.3)	9.2 (8.5 to 10.0)	16.1 (15.0 to 17.2)	48.3 (46.6 to 50.1)	
Maternal smoking status at booking (%, 95% CI)					
Never smoked/quit	57.2 (55.3 to 59.2)	63.0 (61.8 to 64.3)	60.5 (59.0 to 62.0)	50.7 (48.9 to 52.4)	<0.001
Stopped >1 year before conceiving	16.1 (14.6 to 17.5)	17.2 (16.3 to 18.2)	17.7 (16.5 to 18.8)	14.9 (13.7 to 16.2)	
Stopped <1 year prior to conceiving	4.0 (3.3 to 4.8)	2.8 (2.4 to 3.2)	3.5 (3.0 to 4.1)	4.9 (4.2 to 5.7)	
Stopped when pregnancy confirmed	6.8 (5.8 to 7.8)	5.9 (5.3 to 6.6)	6.9 (6.2 to 7.7)	10.3 (9.3 to 11.4)	
Continued smoking	15.9 (14.5 to 17.4)	11.0 (10.2 to 11.8)	11.4 (10.5 to 12.4)	19.1 (17.8 to 20.6)	
Maternal education (%, 95% CI)					
Secondary (GCSE) or under	30.7 (28.9 to 32.5)	24.0 (22.9 to 25.2)	29.4 (28.1 to 30.8)	36.3 (34.6 to 38.0)	<0.001
College (A levels)	40.4 (38.5 to 42.3)	38.8 (37.6 to 40.1)	39.5 (38.1 to 41.0)	45.8 (44.0 to 47.5)	

For peer review only - http://bmjopen.bmj.com/site/about/guidelines.xhtml

University degree or above	28.9 (27.2 to 30.7)	37.1 (35.9 to 38.4)	31.1 (29.7 to 32.5)	17.9 (16.6 to 19.3)	
Maternal employment (%, 95% CI)					
Employed	66.2 (64.3 to 68.0)	71.7 (70.5 to 72.9)	67.2 (65.8 to 68.5)	56.5 (54.8 to 58.2)	<0.001
Unemployed	31.8 (30.0 to 33.7)	26.9 (25.8 to 28.1)	31.1 (29.7 to 32.5)	41.6 (39.8 to 43.3)	
In education	0.9 (0.6 to 1.4)	0.8 (0.6 to 1.1)	1.1 (0.8 to 1.4)	1.3 (0.9 to 1.8)	
Not specified	1.0 (0.7 to 1.5)	0.6 (0.4 to 0.8)	0.7 (0.5 to 1.0)	0.6 (0.4 to 1.0)	
Ethnicity (%, 95% CI)					
White	89.9 (88.7 to 91.1)	88.0 (87.1 to 88.8)	85.1 (84.0 to 86.1)	84.8 (83.5 to 86.1)	<0.001
Mixed	0.8 (0.5 to 1.3)	0.9 (0.7 to 1.2)	1.4 (1.1 to 1.8)	1.6 (1.1 to 2.0)	
Asian	4.8 (4.0 to 5.7)	5.6 (5.0 to 6.0)	7.2 (6.5 to 8.0)	7.7 (6.8 to 8.7)	
Black/African/Caribbean	0.6 (0.4 to 1.0)	1.0 (0.8 to 1.3)	1.6 (1.3 to 2.1)	2.4 (1.9 to 3.0)	
Other	0.7 (0.4 to 1.1)	1.0 (0.8 to 1.3)	1.0 (0.8 to 1.4)	1.3 (0.9 to 1.7)	
Not specified	3.1 (2.5 to 3.9)	3.5 (3.0 to 4.0)	3.6 (3.1 to 4.2)	2.2 (1.8 to 2.8)	
Inter-pregnancy interval (median, IQR)	21.7 (14.4 to 32.7)	21.6 (14.1 to 32.0)	23.7 (14.4 to 35.6)	27.7 (16.0 to 45.6)	<0.001
Inter-pregnancy interval (%, 95% CI)					
0-11 months	17.4 (15.9 to 18.9)	17.6 (16.6 to 18.6)	18.1 (17.0 to 19.3)	16.6 (15.4 to 17.9)	<0.001
12-23 months	39.8 (37.8 to 41.7)	39.9 (38.6 to 41.1)	33.1 (31.7 to 34.5)	26.3 (24.8 to 27.9)	
24-35 months	22.6 (21.0 to 24.2)	23.6 (22.5 to 24.7)	24.4 (23.2 to 25.7)	20.5 (19.1 to 21.9)	
36 months or more	20.3 (18.7 to 21.9)	18.9 (17.9 to 20.0)	24.3 (23.1 to 25.6)	36.5 (34.9 to 38.2)	
Birthweight, grams (mean ± SD)	3463 ± 563	3467 ± 523	3507 ± 536	3531 ± 558	
Previous size at birth (first pregnancy)					
Small-for-gestational age	13.1 (11.8 to 14.4)	12.6 (11.8 to 13.5)	11.7 (10.8 to 12.7)	12.4 (11.3 to 13.6)	0.11
Appropriate-for-gestational age	79.6 (77.9 to 81.1)	81.1 (80.0 to 82.1)	81.2 (80.1 to 82.4)	79.9 (78.4 to 81.3)	
Large-for-gestational age	7.4 (6.4 to 8.5)	6.3 (5.7 to 7.0)	7.1 (6.3 to 7.8)	7.7 (6.8 to 8.7)	
Size at birth (second pregnancy)					
Small-for-gestational age	8.7 (7.6 to 9.8)	7.0 (6.4 to 7.7)	6.2 (5.5 to 6.9)	6.7 (5.9 to 7.6)	<0.001
Appropriate-for-gestational age	79.0 (77.3 to 80.5)	81.1 (80.0 to 82.1)	80.3 (79.1 to 81.5)	77.4 (75.9 to 78.9)	
Large-for-gestational age	12.4 (11.1 to 13.7)	11.9 (11.1 to 12.8)	13.5 (12.5 to 14.5)	15.9 (14.6 to 17.2)	

*p values calculated using ANOVA for continuous and chi square test for categorical variables

For peer review only

For peer review only - http://bmjopen.bmj.com/site/about/guidelines.xhtml

Table 2: Associations between risk of recurrent large-for-gestational age (LGA) birth in the second pregnancy and change in maternal body mass index (BMI) between pregnancies as measured at the first antenatal visit of each pregnancy stratified by BMI category in the first pregnancy

Maternal BMI change (categorised)		Full sample			Normal weight at first pregnancy			Overweight at first pregnancy			Obese at first pregnancy		
		Total n, n of cases	Relative risk, (RR)*	95% CI	Total n, n of cases	RR*	95% CI	Total n, n of cases	RR*	95% CI	Total n, n of cases	RR*	95% CI
Total unadjusted n, n of cases		1109, 530			521, 234			338, 170			236, 122		
Lost ≤ -1 kg/m² from previous pregnancy	Unadjusted	188, 83	0.89	0.74 to 1.08	45, 17	0.80	0.54 to 1.20	74, 30	**0.68**	**0.50 to 0.94**	69, 36	1.16	0.79 to 1.69
	Adjusted**	178, 78	0.88	0.72 to 1.07	44, 16	0.79	0.54 to 1.17	68, 27	**0.69**	**0.48 to 0.97**	66, 35	1.21	0.79 to 1.83
Weight stable (>-1 to <1 kg/m²)	Unadjusted	365, 181	Ref		212, 100	Ref		98, 58	Ref		51, 23	Ref	
	Adjusted**	353, 176	Ref		204, 96	Ref		97, 57	Ref		49, 23	Ref	
Gained 1-3 kg/m² from previous pregnancy	Unadjusted	313, 150	0.97	0.83 to 1.13	162, 74	0.97	0.78 to 1.21	90, 43	0.81	0.62 to 1.06	55, 31	1.25	0.85 to 1.83
	Adjusted**	301, 142	0.98	0.84 to 1.15	156, 70	1.02	0.83 to 1.27	86, 40	0.81	0.61 to 1.08	53, 30	1.28	0.86 to 1.91
Gained ≥3 kg/m² from previous pregnancy	Unadjusted	243, 116	0.96	0.81 to 1.14	102, 43	0.89	0.68 to 1.17	76, 39	0.87	0.66 to 1.14	61, 32	1.16	0.79 to 1.71
	Adjusted**	234, 111	1.00	0.83 to 1.20	96, 39	0.91	0.68 to 1.21	73, 38	0.91	0.67 to 1.25	61, 32	1.28	0.84 to 1.94

*Generalised linear model with log link and robust variance estimator used to derive RR

**Adjusted for: maternal age, ethnicity, highest educational qualification, whether undergone infertility treatment, smoking status, employment status, baseline BMI, gestational diabetes in current pregnancy and inter-pregnancy interval

For peer review only - http://bmjopen.bmj.com/site/about/guidelines.xhtml

Table 3: Associations between the risk of 'new' large-for-gestational age (LGA) birth in the second pregnancy following a non-LGA birth in the first pregnancy and change in maternal body mass index (BMI) between pregnancies measured at the first antenatal visit of each pregnancy stratified by BMI category in the first pregnancy

Maternal BMI change (categorised)		Full sample			Underweight at first pregnancy			Normal weight at first pregnancy			Overweight at first pregnancy			Obese at first pregnancy		
		Total n, n of cases	Relative risk, (RR)*	95% CI	Total n, n of cases	RR*	95% CI	Total n, n of cases	RR*	95% CI	Total n, n of cases	RR*	95% CI	Total n, n of cases	RR*	95% CI
Total unadjusted n, n of cases		14788, 1573			606, 24			8888, 812			3458, 454			1836, 283		
Lost ≤ -1 kg/m^2 from previous pregnancy	Unadjusted	2351, 232	1.05	0.91 to 1.22		-	-	1163, 85	0.88	0.68 to 1.14	690, 79	0.95	0.73 to 1.24	477, 68	0.90	0.67 to 1.23
	Adjusted**	2258, 222	0.94	0.80 to 1.10	-	-	-	1108, 81	0.87	0.68 to 1.12	663, 76	0.96	0.72 to 1.29	466, 65	0.95	0.67 to 1.34
Weight stable (>-1 to <1 kg/m^2)	Unadjusted	5411, 508	Ref		244, 7	Ref		3680, 305	Ref		1024, 123	Ref		463, 73	Ref	
	Adjusted**	5191, 489	Ref		234, 7	Ref		3519, 292	Ref		985, 118	Ref		453, 72	Ref	
Gained 1-3 kg/m^2 from previous pregnancy	Unadjusted	4122, 450	**1.16**	**1.03 to 1.31**	230, 8	1.21	0.45 to 3.29	2606, 259	**1.20**	**1.02 to 1.40**	888, 127	1.19	0.94 to 1.50	398, 56	0.89	0.65 to 1.23
	Adjusted**	3944, 427	1.13	0.99 to 1.28	222, 7	1.04	0.36 to 3.04	2497, 251	**1.26**	**1.06 to 1.50**	839, 115	1.16	0.89 to 1.50	386, 54	0.86	0.61 to 1.22
Gained ≥3 kg/m^2 from previous pregnancy	Unadjusted	2904, 383	**1.40**	**1.24 to 1.59**	111, 9	**2.83**	**1.08 to 7.40**	1439, 163	**1.37**	**1.14 to 1.64**	856, 125	1.22	0.96 to 1.53	498, 86	1.10	0.82 to 1.46
	Adjusted**	2822, 364	**1.34**	**1.17 to 1.54**	104, 6	2.08	0.67 to 6.51	1389, 151	**1.34**	**1.09 to 1.65**	839, 123	**1.35**	**1.05 to 1.75**	490, 84	1.21	0.89 to 1.65

*Generalised linear model with log link and robust variance estimator used to derive RR
**Adjusted for: maternal age, ethnicity, highest educational qualification, whether undergone infertility treatment, smoking status, employment status, baseline BMI, gestational diabetes in current pregnancy and inter-pregnancy interval

For peer review only - http://bmjopen.bmj.com/site/about/guidelines.xhtml

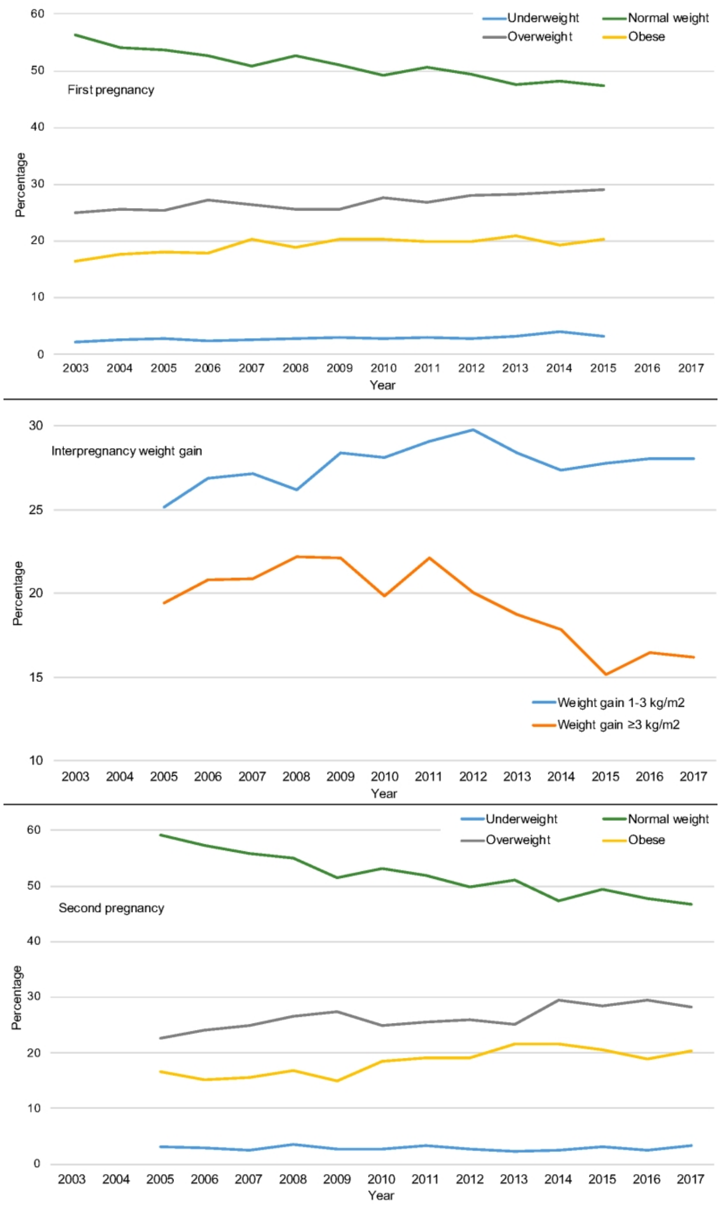

Figure 1: The percentage of women in each body mass index (BMI) category in the first and second pregnancy and weight gain over time in the cohort (2003-2017)

218x367mm (200 x 200 DPI)

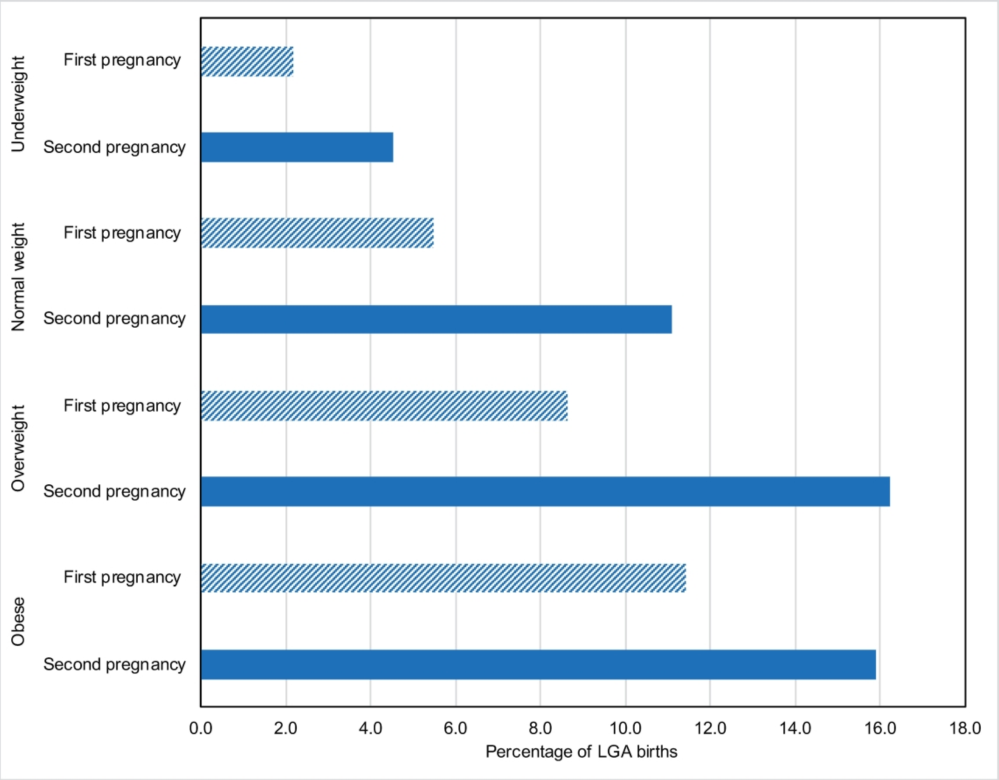

Figure 2: The percentage of large-for-gestational age (LGA) births in first and second pregnancy by maternal body mass index (BMI) category

226x176mm (300 x 300 DPI)

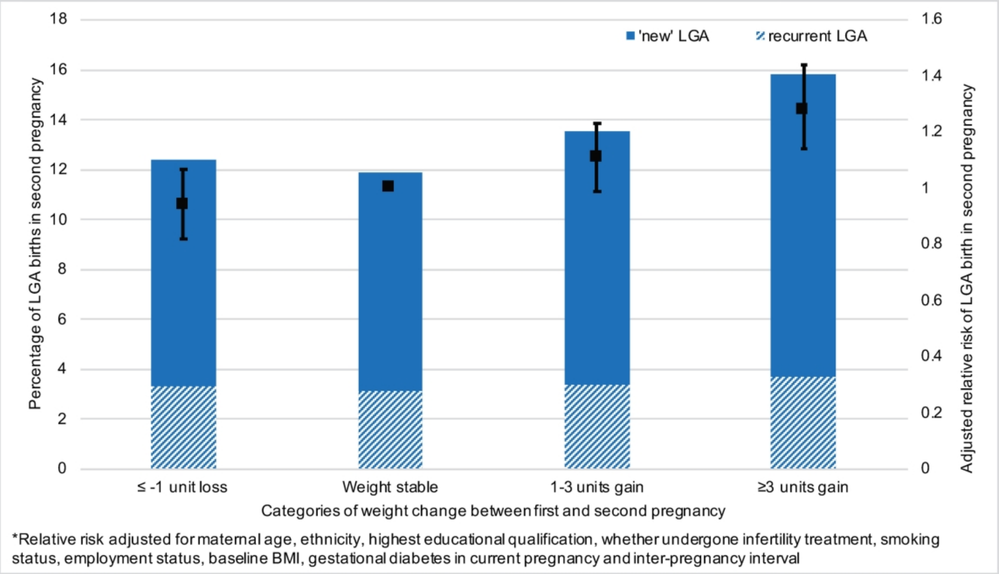

Figure 3: The percentage and risk of large-for-gestational age (LGA) births in second pregnancy stratified by maternal inter-pregnancy weight change categories

222x128mm (300 x 300 DPI)

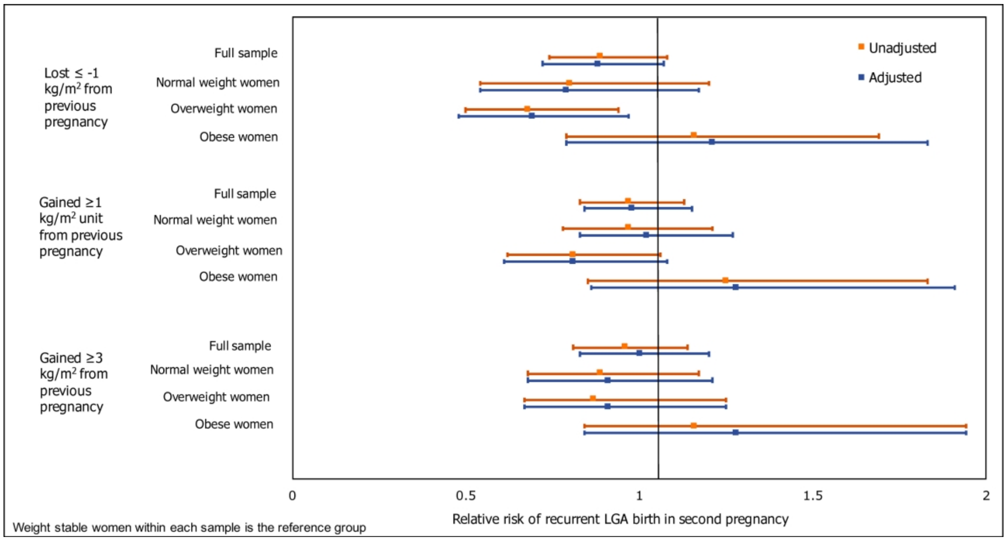

Supplementary Figure 1: Associations between risk of recurrent large-for-gestational age (LGA) birth in the second pregnancy and change in maternal body mass index (BMI) between pregnancies as measured at the first antenatal visit of each pregnancy stratified by BMI category in the first pregnancy

311x165mm (300 x 300 DPI)

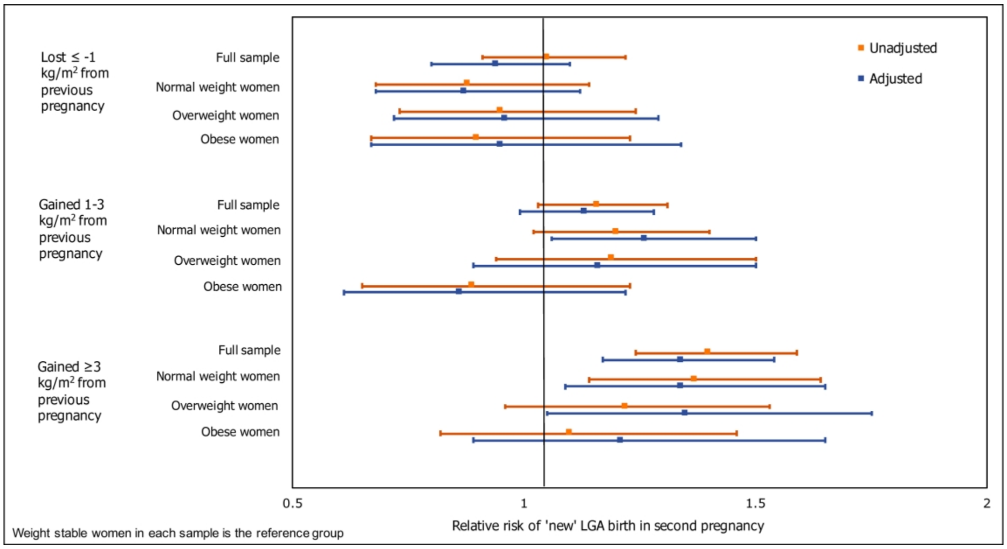

Supplementary Figure 2: Associations between the risk of 'new' large-for-gestational age (LGA) birth in the second pregnancy following a non-LGA birth in the first pregnancy and change in maternal body mass index (BMI) between pregnancies measured at the first antenatal visit of each pregnancy stratified by BMI category in the first pregnancy

310x168mm (300 x 300 DPI)

Reporting checklist for cohort study.

Based on the STROBE cohort guidelines.

Instructions to authors

Complete this checklist by entering the page numbers from your manuscript where readers will find each of the items listed below.

Your article may not currently address all the items on the checklist. Please modify your text to include the missing information. If you are certain that an item does not apply, please write "n/a" and provide a short explanation.

Upload your completed checklist as an extra file when you submit to a journal.

In your methods section, say that you used the STROBE cohort reporting guidelines, and cite them as:

von Elm E, Altman DG, Egger M, Pocock SJ, Gotzsche PC, Vandenbroucke JP. The Strengthening the Reporting of Observational Studies in Epidemiology (STROBE) Statement: guidelines for reporting observational studies.

		Reporting Item	Page Number
Title	#1a	Indicate the study’s design with a commonly used term in the title or the abstract	1 and 2
Abstract	#1b	Provide in the abstract an informative and balanced summary of what was done and what was found	2
Background / rationale	#2	Explain the scientific background and rationale for the investigation being reported	3-4
Objectives	#3	State specific objectives, including any prespecified hypotheses	4
Study design	#4	Present key elements of study design early in the paper	4
Setting	#5	Describe the setting, locations, and relevant dates, including periods of recruitment, exposure, follow-up, and data collection	4
Eligibility criteria	#6a	Give the eligibility criteria, and the sources and methods of selection of participants. Describe methods of follow-up.	4

For peer review only

	#6b	For matched studies, give matching criteria and number of exposed and unexposed	n/a
Variables	#7	Clearly define all outcomes, exposures, predictors, potential confounders, and effect modifiers. Give diagnostic criteria, if applicable	4-5
Data sources / measurement	#8	For each variable of interest give sources of data and details of methods of assessment (measurement). Describe comparability of assessment methods if there is more than one group. Give information separately for for exposed and unexposed groups if applicable.	4-5
Bias	#9	Describe any efforts to address potential sources of bias	n/a
Study size	#10	Explain how the study size was arrived at	4
Quantitative variables	#11	Explain how quantitative variables were handled in the analyses. If applicable, describe which groupings were chosen, and why	4-5
Statistical methods	#12a	Describe all statistical methods, including those used to control for confounding	5
	#12b	Describe any methods used to examine subgroups and interactions	5
	#12c	Explain how missing data were addressed	n/a
	#12d	If applicable, explain how loss to follow-up was addressed	n/a
	#12e	Describe any sensitivity analyses	n/a
Participants	#13a	Report numbers of individuals at each stage of study—eg numbers potentially eligible, examined for eligibility, confirmed eligible, included in the study, completing follow-up, and analysed. Give information separately for for exposed and unexposed groups if applicable.	5
	#13b	Give reasons for non-participation at each stage	n/a
	#13c	Consider use of a flow diagram	n/a
Descriptive data	#14a	Give characteristics of study participants (eg demographic, clinical, social) and information on exposures and potential	5-6

For peer review only

		confounders. Give information separately for exposed and unexposed groups if applicable.	
	#14b	Indicate number of participants with missing data for each variable of interest	13-14
	#14c	Summarise follow-up time (eg, average and total amount)	n/a
Outcome data	#15	Report numbers of outcome events or summary measures over time. Give information separately for exposed and unexposed groups if applicable.	5-6, 13-14
Main results	#16a	Give unadjusted estimates and, if applicable, confounder-adjusted estimates and their precision (eg, 95% confidence interval). Make clear which confounders were adjusted for and why they were included	6-7, 15-21
	#16b	Report category boundaries when continuous variables were categorized	4-5, 13-14
	#16c	If relevant, consider translating estimates of relative risk into absolute risk for a meaningful time period	n/a
Other analyses	#17	Report other analyses done—e.g., analyses of subgroups and interactions, and sensitivity analyses	n/a
Key results	#18	Summarise key results with reference to study objectives	7
Limitations	#19	Discuss limitations of the study, taking into account sources of potential bias or imprecision. Discuss both direction and magnitude of any potential bias.	8-9
Interpretation	#20	Give a cautious overall interpretation considering objectives, limitations, multiplicity of analyses, results from similar studies, and other relevant evidence.	9
Generalisability	#21	Discuss the generalisability (external validity) of the study results	8-9
Funding	#22	Give the source of funding and the role of the funders for the present study and, if applicable, for the original study on which the present article is based	10

The STROBE checklist is distributed under the terms of the Creative Commons Attribution License CC-BY. This checklist was completed on 22. August 2018 using http://www.goodreports.org/, a tool made by the EQUATOR Network in collaboration with Penelope.ai

For peer review only
